# Development of a prediction system for tail-anchored proteins

**DOI:** 10.1186/s12859-016-1202-7

**Published:** 2016-09-15

**Authors:** Shunsuke Shigemitsu, Wei Cao, Tohru Terada, Kentaro Shimizu

**Affiliations:** 1 Department of Biotechnology, Graduate School of Agricultural and Life Sciences, The University of Tokyo, 1-1-1 Yayoi, Bunkyo-ku, Tokyo, 113-8657 Japan; 2 Agricultural Bioinformatics Research Unit, Graduate School of Agricultural and Life Sciences, The University of Tokyo, 1-1-1 Yayoi, Bunkyo-ku, Tokyo, 113-8657 Japan

**Keywords:** TA proteins, HMMs, Membrane proteins, Prediction, Machine learning

## Abstract

**Background:**

“Tail-anchored (TA) proteins” is a collective term for transmembrane proteins with a C-terminal transmembrane domain (TMD) and without an N-terminal signal sequence. TA proteins account for approximately 3–5 % of all transmembrane proteins that mediate membrane fusion, regulation of apoptosis, and vesicular transport. The combined use of TMD and signal sequence prediction tools is typically required to predict TA proteins.

**Results:**

Here we developed a prediction system named TAPPM that predicted TA proteins solely from target amino acid sequences according to the knowledge of the sequence features of TMDs and the peripheral regions of TA proteins. Manually curated TA proteins were collected from published literature. We constructed hidden markov models of TA proteins as well as three different types of transmembrane proteins with similar structures and compared their likelihoods as TA proteins.

**Conclusions:**

Using the HMM models, we achieved high prediction accuracy; area under the receiver operator curve values reaching 0.963. A command line tool written in Python is available at https://github.com/davecao/tappm_cli.

**Electronic supplementary material:**

The online version of this article (doi:10.1186/s12859-016-1202-7) contains supplementary material, which is available to authorized users.

## Background

“Tail-anchored (TA) proteins” is a collective term for transmembrane proteins with a C-terminal transmembrane domain (TMD) and without an N-terminal signal sequence. They account for approximately 3–5 % of all transmembrane proteins that are involved in a wide range of processes such as membrane fusion, regulation of apoptosis, and vesicular transport [1]. Because TA proteins lack N-terminal signal sequences, they are not recognized by the ribosomal signal recognition particle (SRP). After translation, they are bound by an ATPase (TRC40 in mammals and Get3 in yeast) at their TMD [2, 3]. After binding to ATP, TRC40 and Get3 form a so-called “closed” structure, and the resulting hydrophobic groove recognizes the TMD of TA proteins [4–8].

The research on TA proteins started with synaptobrevin, a soluble N-ethylmaleimide-sensitive factor (NSF) attachment protein receptor (SNARE). Certain membrane proteins with a single TMD near the C-terminus are not recognized by SRP, because their TMD remains within the ribosome when translation terminates [9]. Kutay et al. found that synaptobrevin does not interact with SRP or SEC61 during its translocation to the membrane [10]. Examples of TA proteins include SNARE proteins involved in membrane fusion, Bcl-family proteins that regulate apoptosis, and the electron transport chain component cytochrome b5.

Although the mechanism of translocation of TA proteins is gradually being unraveled, more research is required along with new tools that can predict a TA protein from its amino acid sequence. However, public-domain TA-protein prediction tools are not available. Furthermore, it is difficult to predict TA proteins according to amino acid sequence similarities, because they belong to different families that share only their C-terminal TMDs.

Numerous methods for identifying transmembrane proteins were either proposed or are available as web-based tools, and many are very accurate [11–13]. However, no publicly available prediction tool can distinguish TA proteins from other types of membrane proteins. The conventional computational method to predict TA proteins analyzes and integrates the results obtained from multiple tools [14]. Such procedures specifically require TMD prediction tools such as TMHMM and signal sequence prediction tools such as SignalP and TargetP [15–18]. To partially circumvent such cumbersome procedures and to improve prediction accuracy, a dedicated prediction tool capable of distinguishing TA proteins from other membrane proteins is expected to be highly useful. To address this issue, we developed a machine-learning based technique to predict TA proteins from only target amino acid sequences, which employs Hidden Markov Models (HMMs). The high-speed prediction capabilities of this tool can be used to analyze an entire genome.

## Results and discussion

### Likelihood score predictions

The results of likelihood score-based predictions are shown in Table 1. For predictions with the signal peptide (SP) and TA datasets, the scores were calculated from the likelihood values of the TA and SP models. For predictions with the membrane protein (MP) dataset, the nonmembrane protein (NO) set, all negative data, and the TA dataset, scores were calculated from likelihood values of the TA and MP models. We chose values of the threshold scores that minimized the balanced error rate (BER) (see ‘Methods’).

**Table 1:** Likelihood score classification

Negative data	AUC	Sensitivity	Specificity	TP	FP	FN	TN
SP	0.957	0.877	0.917	142	108	20	1199
MP	0.971	0.901	0.934	146	27	16	383
NO	0.967	0.895	0.929	145	355	17	4675
Total	0.963	0.864	0.952	140	327	22	6420

Each row corresponds to the specified individual negative data. The “total” at the bottom of the table shows the cross-validation results for all negative data. TP, FP, FN, and TN represent true-positive, false-positive, false-negative, and true-negative values, respectively

The discriminatory powers of the SP and TA datasets were indicated by sensitivity and specificity values of approximately 0.877 and 0.917, respectively, and those of the MP and TA datasets were approximately 0.901 and 0.934, respectively. Thus, the latter achieved the best performance among the three groups. The sensitivity and specificity of the NO set were 0.895 and 0.929, respectively.

To discriminate between the all negative and TA datasets, sensitivity was 0.864 (0.013-0.037 points lower than using individual discrimination). Because of this reduced ability to discriminate between the datasets, the number of sequences that were correctly recognized as TA proteins decreased by 2. In contrast, specificity was approximately 0.952, which was higher than that of any other example of individual discrimination. The number of false positives was 327, which was fewer than that of individual discrimination using the NO set.

**Fig. 1:**
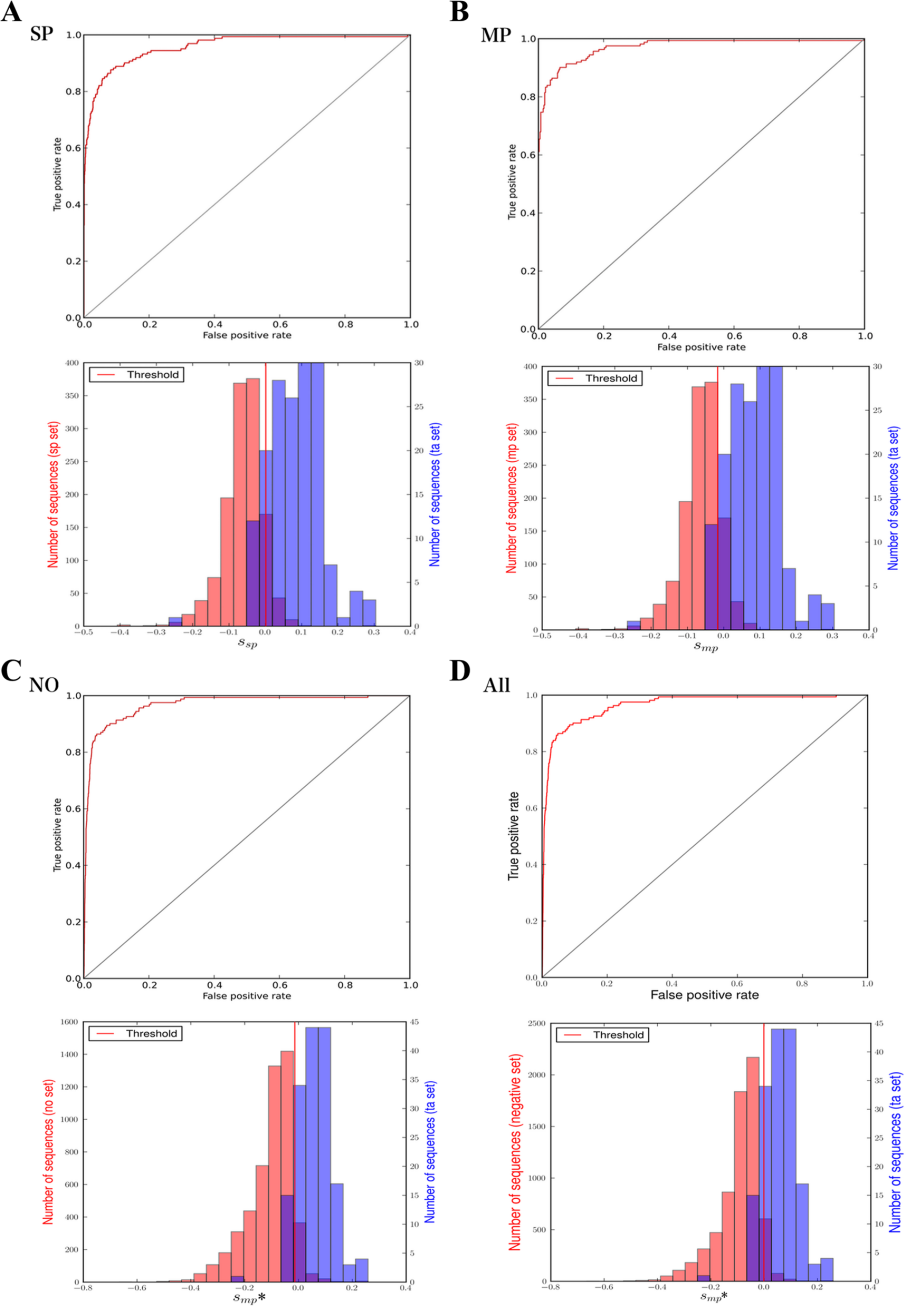
Results of likelihood score-based prediction. In **a** to **d**, the upper graphs show the ROC curves, and the lower graphs shows the histogram of likelihood scores. The vertical red lines represent the thresholds for calculating sensitivity and specificity. For NO and ALL set, the MP scores are used as their likelihood scores (denoted as Smp*) and the threshold value of the MP dataset is used as their threshold values

Receiver operating characteristic (ROC) curves and likelihood distributions from discrimination using the SP, MP, NO, and all-negative datasets are shown in Fig. 1. In all four cases, the distributions of the likelihood scores were not completely distinguishable between the positive and negative data. The distribution of the likelihood scores of the negative data peaked at a single value, whereas there were two or three peaks in the distribution of the TA sets.

Although almost all likelihood scores in the TA set were > –0.1, the scores were < –0.2 for only one sequence (UniProt ID: YD012_YEAST). With this exception, the minimum likelihood scores of the TA set were −0.048 in *S*_*sp*_ (score for the SP model) and −0.056 in *S*_*mp*_ (score for the MP model).

### Decoding-based predictions

The decoding-based prediction results are shown in Table 2. Because the determination is based on the presence or absence of TMD and tail regions, this prediction method lacks a threshold. Therefore, sensitivity and specificity were used as the evaluation indices.

**Table 2:** Classification by decoding

Name	Sensitivity	Specificity	TP	FP	FN	TN
SP	0.901	0.881	146	153	16	1154
MP	0.901	0.924	146	23	16	387
NO	0.901	0.960	146	199	16	4831
Total	0.901	0.944	146	375	16	6372

Each row corresponds to the specified individual negative data. The “total” at the bottom of the table shows the cross-validation results of all negative data. The area under the receiver operator curve (AUC) cannot be calculated because there are no indices (e.g., likelihood scores) associated with this method

**Fig. 2:**
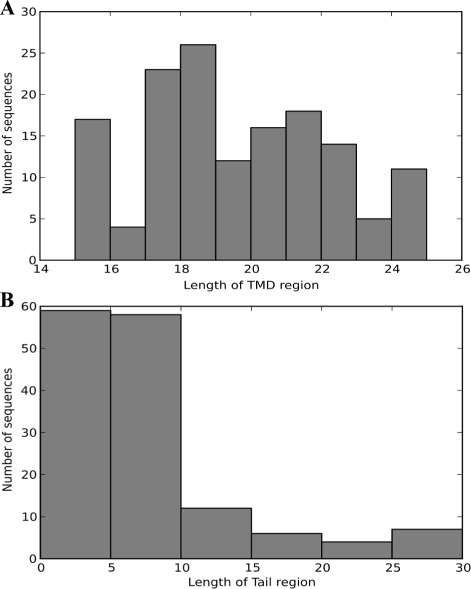
Distribution of predicted lengths of tail and TMD regions. **a** Histogram of the predicted lengths of the TMD regions in sequences successfully predicted to have TMD and tail regions (146 sequences). **b** Histogram of the predicted lengths of the tail regions in sequences successfully predicted to have TMD and tail regions (146 sequences)

In the TA set, TMD and tail regions were correctly predicted for 146 proteins, although prediction attempts failed for 16 proteins. Of the proteins that were not predicted, nine had no TMD region in the decoded sequence, and seven had a tail region comprising at least 31 residues and a TMD region. For the 146 sequences with successfully predicted tail regions, the lengths of the predicted TMD and tail regions are shown in Fig. 2.

Because of the structural constraints of the TA model, the lengths of TMD regions were maintained between 15 and 25 residues. The most abundant sequences (27 sequences) were those with predicted lengths of 18 residues, followed by 24 sequences predicted with 17 residues and 18 sequences predicted with 21 residues. Of the 146 successful predictions, 124 possessed tail regions comprising ≤10 residues, accounting for 84.9 % of the successfully predicted sequences and 76.5 % of all TA-set sequences. In contrast, 22 sequences were predicted with tail regions ≥11 residues. Among the sequences with failed tail-region predictions but successful TMD-region predictions, the shortest tail region was 31 residues (2 sequences), and the longest was 92 residues.

### Predictions using both likelihood score and decoding

The results of predictions combining the likelihood score-based method and the decoding-based method are shown in Table 3. Only sequences judged as TA proteins by both methods were regarded as positive. Because no threshold exists for this method (as in the decoding-only method), the evaluation was based on sensitivity and specificity.

**Table 3:** Predictions using likelihood scores and decoding

Name	Sensitivity	Specificity	TP	FP	FN	TN
SP	0.846	0.947	137	69	25	1238
MP	0.870	0.956	141	18	21	392
NO	0.920	0.980	149	101	22	4929
Total	0.852	0.978	138	151	24	6596

Each row corresponds to the specified individual negative data. The “total” at the bottom of the table shows cross-validation results of all negative data. The area under the receiver operator curve (AUC) cannot be calculated, because there are no indices (e.g., likelihood scores) associated with this method

Compared with predictions based only on likelihood scores or only decoding, sensitivity decreased while specificity increased using this method, because the results were the product of the predictions of both methods. Although sensitivity ranged from approximately 85 % to a maximum of 92 %, specificity remained between approximately 94–98 %. This high specificity may prove useful in applying the method to genomes with all-negative data.

### Discussion

In the present study, we used likelihood score-based predictions to achieve AUC values ≥0.95 for every dataset, indicating that HMMs developed here successfully reflect the characteristics of TA proteins and membrane proteins with signal sequences. The MP dataset had the highest AUC, likely because the topologies of its members, which possess multiple TMDs, differ significantly from those of TA proteins. Many proteins included in the NO dataset were also expected to have structures different from those of TA proteins and they did not share common features except for signal sequences, and had no dedicated models. Consequently, the AUC value for the NO set was slightly lower than that of the MP set. Further, the SP set had the lowest AUC. In this set, 198 sequences contained TMDs within 30 residues of the C-terminus (approximately 15 % of all sequences in the SP set). With so many structurally similar sequences, discrimination from the TA set would be more difficult than that with the other two datasets.

In all methods, we identified 10 common cases of failed predictions (Additional file 1: Table S5). Of the sequences with failed predictions, the SEC20_YEAST sequence encodes a SNARE protein, which is involved in transport in the Golgi apparatus, and forms a complex with UFE1 and USE1 [19]. This protein contains a TMD, but its end is situated 91 residues distal to the C-terminus. As described in the ‘Methods’ section, the majority of TA TMD domains reside within 10 residues of the C-terminus; therefore, in the present model, a TMD 91 residue away from the C-terminus will not be detected and thereby failed to be predicted. The YD012_YEAST sequence is another example of a failed prediction. Although the function of this sequence is unknown, it localizes to the plasma membrane [20]. The sequence contains glutamine (Q)-rich regions, accounting for its low hydrophobicity and low likelihood score (Additional file 2: Figure S2).

To further investigate the characteristics of unsuccessfully predicted sequences, we analyzed the lengths and hydrophobicity values of the sequences listed at least once in Table S5. The length of unsuccessfully predicted sequences ranged from <100 residues to approximately 1000 residues (Additional file 3: Figure S3). However, in the 200–300 residue sequence range that includes an abundance of TA proteins, only one sequence was not predicted (UniProt ID: O80952_ARATH). In contrast, the failure rate was relatively high for proteins with long sequences. Thus, 9 of 30 TA proteins with ≥400 residues were not predicted (30 % failure rate). Likewise, of the 23 TA proteins <100 residues, eight were not predicted.

These results may be attributable to the Baum–Welch algorithm used for training HMMs, which is a type of maximum likelihood estimation, and depends on the training data. Therefore, the models were highly compatible with more accurately annotated 200–300 residue sequences. However, because data is scarce for sequences of vastly different lengths, the characteristics of such sequences were likely not fully reflected during training. The hydrophobicity scores for many individual sequences of TA proteins were highest 10–20 residues upstream of the C-terminus, thereby creating highly hydrophobic regions near the C-terminus. Nevertheless, in some sequences, hydrophobic regions were relatively distant from the C-terminus (e.g., NDB3B_ARATH, TO221_ARATH, and MTX1_HUMAN). Moreover, because SEC20_YEAST and TLG2_YEAST sequences possess TMDs >50 residues upstream from the C-terminus, there were no hydrophobic regions in these plots. In such sequences, the likelihood decreased during decoding until a TMD was detected. Therefore, predicting their correct topology may be more difficult than for sequences with hydrophobic domains in the proximity of the C-terminus.

For the genomes of humans, yeast, and thale cress, the conventional prediction method involves the combined use of existing prediction tools for TMDs and signal sequences.

The conventional method employed the tools TMHMM [16] to predict TMDs and SignalP [15] to predict signal sequences. We applied this method to the TA set to determine its differences with our method named Tailed-Anchored Protein Prediction Method, TAPPM (Table 4).

**Table 4:** Sequences not predicted by the TMHMM or our method TAPPM

TAPPM	TMHMM	TMHMM	TAPPM
failures	failures	failures and	failures and
		TAPPM success	TMHHM successes
TOM7_YEAST	GDAP1_HUMAN	TOM22_YEAST	O22825_ARATH
PGC1_YEAST	PEX15_YEAST	YBM6_YEAST	MAVS_HUMAN
TOM7_HUMAN	PGC1_YEAST	GDAP1_HUMAN	TOM6_YEAST
MAVS_HUMAN	SEC20_YEAST	PEX15_YEAST	TLG2_YEAST
O22825_ARATH	TOM22_YEAST	VPS64_YEAST	TOM7_HUMAN
GEX2_ARATH	TOM7_YEAST	UFE1_YEAST	GEX2_ARATH
MTX1_HUMAN	UFE1_YEAST		MTX1_HUMAN
YD012_YEAST	VPS64_YEAST		Q9FNB2_ARATH
TOM6_YEAST	YBM6_YEAST		
TLG2_YEAST	YD012_YEAST		
Q9FNB2_ARATH			
SEC20_YEAST			

The TMHMM/SignalP and the TAPPM methods failed to predict 10 and 12 TA proteins, respectively. The prediction rate of SignalP was 100 %. To construct the TA dataset, we used annotations from the UniProt/SwissProt database that assigns signal sequence annotations according to SignalP. Therefore, our datasets contained only sequences that were successfully predicted using SignalP. Although we did not necessarily consider this while compiling our datasets, the TMD annotations were partially based on TMHMM. Because datasets contained pre-existing findings derived from the conventional method, this may impart a slight advantage over our method for accurate predictions. Although our method failed to predict more sequences than the conventional method, some successful predictions included those missed by the conventional method. In particular, ganglioside-induced differentiation-associated protein 1 (UniProt ID: GDAP1_HUMAN) is an empirically confirmed TA protein, and UniProt/SwissProt assumes it has two TMDs [21]. Therefore, we believe that the significance of our method lies in its ability to predict sequences that are not predicted by the conventional method and for sequences in databases with annotation errors.

## Conclusions

In the present study, we developed a prediction system, TAPPM, to identify TA proteins from amino acid sequences using HMMs, which provides highly accurate predictions. By taking advantage of the properties of HMMs, we devised two evaluation methods with varying characteristics. Further, we collected data from empirically confirmed TA proteins to include in the training data. Despite reduced accuracy to a certain extent compared with that of the conventional method, our method predicted the sequences of a few TA proteins that were not predicted using the conventional method. Another advantage of our method is that it avoids the cumbersome procedures required to use the conventional methods.

We will aim to achieve even higher specificity, which is required for predicting genomic sequences that encode TA proteins. To achieve this goal, we will optimize HMMs for TA proteins and construct sequence models for non-TA proteins. The identities of intracellular organelles harboring TA proteins are unknown as well as the motifs and mechanisms required for their transport. Therefore, we believe that prediction of subcellular localization will contribute to identifying the transport mechanisms.

## Methods

### Datasets

Before constructing a prediction tool using machine learning, amino acid sequence data for TA and non-TA proteins with different signal sequences were prepared as positive and negative datasets, respectively.

#### Positive dataset

We manually curated TA protein data mainly from published literature for studying TA proteins in humans (*Homo sapiens*), thale cress (*Arabidopsis thaliana*), and budding yeast (*Saccharomyces cerevisiae*) [14, 22, 23].

To exclude the non-experimentally confirmed TA proteins, we searched initially collected TA proteins against the existing databases. The following databases were used: UniProt (The UniProt Consortium 2012), Saccharomyces Genome Database [24], TAIR [25], and SUBA 3 [26]. The dataset initially contained 190 sequences; however, 22 sequences are either empirically unsubstantiated or are nonmembrane proteins and were therefore excluded from the dataset, leaving 168 sequences (Additional file 4: Table S1). To eliminate sequence redundancy within the dataset, clustering was performed using BLASTClust [27] with an 80 % sequence identity threshold and 80 % length coverage. As a result, 162 clusters were generated, and their representative 162 sequences were selected as the final version of the TA protein dataset (Table 5). The condition of this clustering is not common for general reductions in homology. However, TA proteins have the unique characteristics at the C-terminus of the amino acid sequences which are responsible for targeting membranes of different organelles. We used the above condition to avoid the removal of relatively similar sequences but whose C-terminus subsequences are different. In our analysis of these 162 sequences, all but four sequences actually had pairwise sequence identities less than 20 % in the C-terminal subsequences of 30 amino acids, i.e., the parts subjected to the prediction, demonstrating that our method could produce a dataset containing a wide variety of sequences.

**Table 5:** Subcellular locations of the collected TA protein sequences

Subcellular location	# of sequences
Endoplasmic reticulum	52
Plasma membrane	46
Golgi apparatus	33
Plastid	13
Nucleus	7
Vacuole	8
Peroxisome	3
Synaptic vesicle membrane	3

Certain sequences localize to several subcellular locations; therefore, the sum is <162

Table 5 shows the numbers of sequences localized to subcellular compartments. While the majority localize to the endoplasmic reticulum (ER), plasma membrane, and Golgi apparatus, some reside in vacuoles or peroxisomes.

#### Negative dataset

We collected non-TA protein data from the UniProt/SwissProt database. The annotation (features) section of each sequence was analyzed to determine the presence or absence of signal sequences, and the number of TMDs. Apart from signals belonging to secretory proteins, signals for localization to the nuclear membrane, mitochondria, and plastids were regarded as equivalent to a signal sequence. Only those TMDs in the database with the description “helical” were used to facilitate efficient learning of the difference between TA and non-TA proteins. All data entries had a signal sequence and were divided into the groups as follows:

1. Multiple TMDs (multi-pass, “MP set”)
2. Single TMD (single-pass, “SP set”)
3. No TMD (nontransmembrane, “NO set”)

The MP, SP, and NO sets comprised 1902, 5779, and 26,554 sequences, respectively. To eliminate redundancy, clustering was performed using BLASTClust, with clustering conditions of 40 % length coverage and 45 % score coverage. From each generated cluster, one sequence was randomly chosen and included in the final dataset, and the final MP, SP, and NO datasets contained 410, 1307, and 5030 sequences, respectively.

### HMMs

In the present study, we constructed three HMMs corresponding to the datasets as follows: the TA model for the TA structure in the positive dataset, the SP model for the SP dataset structure in the negative dataset, and the MP model for the MP dataset structure in the negative dataset. Descriptions of the respective models are presented below.

#### TA model

**Fig. 3:**
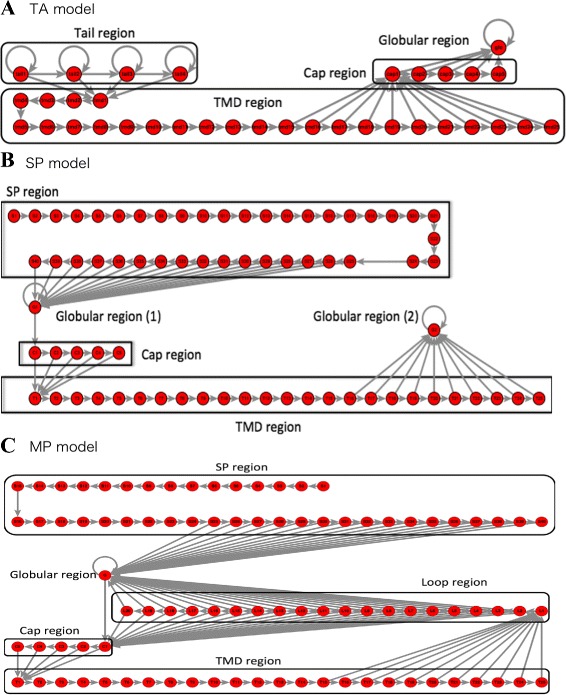
Transition-state diagram of HMM models. **a** TA model: Circles represent nodes (hidden states) and arrows signify transitions. Two models were constructed with either two or four states in the tail region. **b** SP model: Although globular region (1) and globular region (2) have different transitional states, their initial output probability conditions were identical. **c** MP model: Under the initial conditions, the output and transition probabilities of the SP, cap, and TMD regions were identical to those of the SP model. The loop region may comprise one to 20 residues

The entire structure of the TA model can be approximately divided into four domains, which we refer to (from the N-terminus) as the globular, cap, TMD, and tail regions (Fig. 3a). The starting point was placed at the C-terminus of the sequence so that the TMDs of TA proteins, which were mainly distributed near the C-terminus, could be easily recognized. If many transitions were required for the HMM before reaching the TMD, the probability of detecting a TMD was extremely low. Before training, the output probability of each region was defined (Additional file 5: Table S2). Further, the initial probabilities of the tail and TMD regions in the initial state were each defined as 50 %. Output probabilities were set such that hydrophilic, hydrophobic, and basic residues were more likely to occur in the tail, TMD, and cap regions, respectively. The definition of these amino acid groups were given in Additional file 5: Table S2.

The starting point of this model is a hydrophilic site at the end of the C-terminus (tail region). The tail region has the transition states as follows: itself, the adjacent state, and the initial state of the TMD. The initial transition probabilities were set such that all transition states were equally probable. For the initial output probability conditions, hydrophilic residues were allowed to occur more frequently. The number of states was set to either two or four, and both were used for the calculations of decoding and probability (described later).

The modeled TMD region resides next to the tail region. This region consists of 25 transition states, and the first 14 states transition only to the next state. This design ensured that the TMD was at least 15 residues long. The 11 states that follow transition to the next state and to the initial state in the cap region. The initial output probability conditions were set such that hydrophobic residues were more likely to occur, and the hydrophilic and basic residues were less likely to occur.

In the cap region, basic residues were concentrated in the proximity of TMD. The cap region was assumed to retain TMD within the plasma membrane through interactions between lipids and hydrophilic residues [28]. The cap region was designed with a maximum length of five residues, with two transition states, the next state and hydrophilic states. The initial output probability conditions for this region were set such that basic residues were abundant. Finally, the modeled hydrophilic domain, which was the sequence closest to the N-terminus, was located at the N-terminus of the protein (globular region). This region has only one state capable of transitioning to itself.

#### SP model

The SP model encompasses modeled membrane proteins with a signal sequence and a single TMD. The objective of this model was to train the HMM to recognize the signal sequences in TMD and those near the N-terminus, thereby distinguishing between the TA and the SP proteins that are structurally similar to TA proteins. The initial output probability conditions for this model are presented in Additional file 6: Table S3. The transition-state diagram for this model is shown in Fig. 3b. The starting point of this model was the N-terminal amino acid residue. We assigned a probability of 50 % to each of the initial states of the signal peptide and globular regions, and the probability of the other states was set to 0 %. Similar to the TA model, the SP model can be divided approximately into sections referred to (from the N-terminus) as the signal peptide (SP) region, globular region (1), cap region, TMD region, and globular region (2).

The signal peptide region is situated at the front of the model and recognizes protein signal sequences. In general, signal sequences are approximately 15 to 30 residues long and there is no definite consensus sequence. Nevertheless, a typical signal sequence can be broadly divided into three regions with different characteristics [29]. At the N-terminal end, there are frequent occurrences of many positively charged residues. Adjacent is a region comprising ≥6 hydrophobic amino acid residues. At the C-terminal end, uncharged polar residues frequently occur with the conservation of one or three residues away from the signal-sequence cleavage site, and the protein is transferred from the translocon toward the N-terminus after cleavage.

To reflect the characteristics of these three sections in our model signal peptide region, we attempted to reproduce the aforementioned differences by altering the initial output probabilities of the first 10 residues and the two downstream regions comprising 15 residues each. Basic, hydrophobic, or hydrophilic residues were specifically set to occur more frequently in the first 10 and the two subsequent 15 residue regions, respectively.

The region contiguous to the signal peptide region is the globular region (1), which was the modeled domain present between the signal sequence and TMD. This region has itself and the next cap region as its transition states.

Similar to the TA model, the cap region models the area near TMD where basic residues are concentrated. This region was designed with one to five residues and a higher representation of basic residues in their initial conditions. The subsequent TMD region is the modeled TMD with the same structure as its counterpart in the TA model and was designed to include between 15 and 25 residues. Therefore, the first 15 states can only transition to the next state, whereas the next 10 states can transition to the globular region (2) and the next state. Located at the end, the globular region (2) is the modeled hydrophilic domain located outside TMD, and its single transition state is itself. The other probability parameters were the same as those in the globular region (2).

#### MP model

The MP model encompasses proteins with a signal sequence and multiple TMDs. This model was constructed with the aim of establishing a general-purpose archetype applicable to all membrane proteins with a signal sequence. The transition-state diagram for this model is shown in Fig. 3c. The MP model can be approximately divided into 5 sections, of which the signal peptide, globular, cap, and TMD regions are similar to those of the SP model. The additional region is the loop region, which is a modeled looping structure between TMDs. Although the overall structure is similar to that of the SP model, a marked difference is that the TMD region is followed by a loop region instead of the globular region, and this loop region can transition back to the cap region.

The starting point of the amino acid sequence was also the N-terminal amino acid residue. The model was designed so that the signal peptide and globular regions had initial probabilities of 50 %, and the initial probabilities of the other states were set to zero. Under initial conditions, the structures of the signal peptide, globular, cap, and TMD regions were identical to those of the SP model. The loop region follows the TMD region. Based on a report that the loop section linking the TMD consists of no more than approximately 20 residues [30], this region was 1–20 residues long. Because two TMDs were not always linked by a looping structure, each state in the loop region can transition to the globular region in addition to the cap region. Under the initial conditions, transitions to the next state within the loop, globular, and cap regions were set to occur with equal probability. The initial output probability conditions (Additional file 7: Table S4) within the loop region were set such that hydrophilic residues were more likely to occur.

#### Training and evaluation

We implemented two algorithms in python, the Baum-Welch learning algorithm to train our HMM models, and the Viterbi decoding algorithm [31] to detect the C-terminal TMD and obtain models’ likelihoods. In training procedure, we used a 5-fold cross validation to obtain the optimal parameters of HMM models. Concretely, for every sequence in the datasets, decoding was performed to obtain their likelihoods using the respective TA, SP, and MP models. We acquired three likelihoods and the most probable hidden sequence per protein sequence to establish the evaluation criteria.

One evaluation method used a sequence of regions (defined as the state sequence) in which each residue in the sequence belonged to the sequence with the highest likelihood. The TA model included the regions (states) as follows: TMD, tail, cap, and globular; and each of the state sequence was one of the four states. With this method, the only criterion was whether or not a TMD or tail region of appropriate length was contained within the decoded state sequence. A sequence was specifically identified as a TA protein if it had a TMD region comprising ≥15 residues and a tail region ≤30 residues. For decoding, we used the TA model with two loop sections within the tail region. If it was decoded as a tail region over its entire length, no transition to the TMD region occurred (Additional file 8: Figure S1).

The second evaluation method compared the likelihood of the different models inferred from the Viterbi algorithm. For specific sequences, comparison of the likelihoods obtained from different models enabled selection of the model that best represented the sequence. For example, if the likelihood value of the TA model was higher than that of the SP model for a certain sequence, in comparison, that sequence was assumed more structurally similar to the TA model than the SP model. Thus, this approach can be used to determine whether the sequences represent TA proteins. The scores used for comparisons were calculated as the difference between the log value of the likelihood of the SP or MP model (*lnl*_*sp*_, *lnl*_*mp*_) and that of the TA model (*lnl*_*ta*_) divided by the length of the sequence. The scores of SP model and MP model are denoted as *S*_*sp*_ and *S*_*mp*_, respectively. Because the log-likelihood values were calculated by adding the logs of the probabilities, longer sequences computed to smaller values.

Therefore, longer sequences had larger absolute log-likelihood values, and normalization was achieved to a certain extent by dividing by the sequence length. After calculating the score for each sequence, we determined a threshold value for discriminating the different datasets with the highest efficacy. The threshold value was defined as the value with the smallest balanced error rate (BER). The indices used were as follows:

$$\begin{array}{@{}rcl@{}} &Accuracy =\frac{TP + TN}{TP+FP+FN+TN}\\ &Sensitivity =\frac{TP}{TP+FN} \\ &Specificity =\frac{TN}{FP+TN} \\ &BER =\left(\frac{FN}{TP+FN} + \frac{FP}{FP+TN}\right)\div 2 \end{array} $$

Here TP, FP, FN, and TN signify true-positive, false-positive, false-negative, and true-negative values, respectively.

The methods based on the decoding results and the likelihood values were combined in the present study, and the sequences that scored positive using both methods were defined as TA proteins. For each X of the three datasets TA, SP, and MP, the likelihood of each X of three protein models TA, SP, and MP was calculated. For dataset X, cross-validation was only performed to calculate the likelihood of corresponding model X and all data were used (cross-validation was not performed) for the other models. Because data from other models were not used for training, there was no risk of overtraining caused by the use of the same data for training and evaluation. The log-ratio of likelihoods of TA and MP are taken as the prediction score in the web application.

## Additional files

Additional file 1
**Table S5.** Sequences that were not predicted. (PDF 40 kb)

Additional file 2
**Figure S2.** YD012_YEAST sequence and hydrophobicity scores. (A): Amino acid sequence of YD012_YEAST, which is exceptionally rich in glutamine (Q). (B): Distribution of hydrophobicity scores. Linear smoothing was applied to the Kyte-Doolittle hydrophobicity indices. (TIF 162 kb)

Additional file 3
**Figure S3.** Lengths and hydrophobicities of sequences that were not predicted. Upper: Sequence lengths. The gray bars represent all TA proteins and red portions indicating prediction failures. Lower: Hydrophobicity scores of unsuccessfully predicted sequences. Linear smoothing was applied to Kyte–Doolittle hydrophobicity scores. Each row corresponds to a sequence, and each column represents a residue. The sequences are listed in order of hydrophobicity between the C-terminus and the 50th residue (sequences in the upper rows are more hydrophobic). (TIF 1658 kb)

Additional file 4
**Table S1.** Empirically validated TA proteins. (PDF 119 kb)

Additional file 5
**Table S2.** Initial conditional probability in the TA model. (PDF 35 kb)

Additional file 6
**Table S3.** Initial conditions for the SP model. (PDF 35 kb)

Additional file 7
**Table S4.** Initial conditions for the MP model. (PDF 37 kb)

Additional file 8
**Figure S1.** Decoding example. Decoding using the TA model for human cytochrome b5 and yeast TOM7. The first line is the header describing details such as the sequence name. From the second line onward, the amino acid sequence (Seq) and decoded state sequence (Path) appear alternately with direct correspondence between the amino acid residues and state designations. In state sequences, the letters G, C, H, and T indicate the globular, cap, TMD (hydrophobic), and tail regions, respectively. In the example shown, a TMD (H) was predicted in the proximity of the C-terminus of cytochrome b5 (success), whereas the entire TOM7 sequence was predicted to be a tail region (failure). (TIF 864 kb)

## Abbreviations

AUC: Area under the receiver operator curve
BER: Balanced error rate
CLI: Command line interface
FN: False-negative
FP: False-positive
HMM: Hidden Markov model
SNARE: Soluble N-ethylmaleimide-sensitive factor attachment protein receptor
SRP: Signal recognition particle
TA proteins: Tail-anchored proteins
TMD: Transmembrane domain
TN: True-negative
TP: True-positive
FN: False-negative
FP: False-positive

## References

[1] Hegde RS, Keenan RJ. Tail-anchored membrane protein insertion into the endoplasmic reticulum. Nat Rev Mol Cell Biol. 2011; 12(12):787–98. doi:http://dx.doi.org/10.1038/nrm3226. 10.1038/nrm3226 PMC3760496 22086371

[2] Schuldiner M, Metz J, Schmid V, Denic V, Rakwalska M, Schmitt HD, Schwappach B, Weissman JS. The GET complex mediates insertion of tail-anchored proteins into the ER membrane. Cell. 2008; 134(4):634–45. doi:http://dx.doi.org/10.1016/j.cell.2008.06.025. 10.1016/j.cell.2008.06.025 PMC2572727 18724936

[3] Stefanovic S, Hegde RS. Identification of a targeting factor for posttranslational membrane protein insertion into the ER. Cell. 2007; 128(6):1147–59. doi:http://dx.doi.org/10.1016/j.cell.2007.01.036. 10.1016/j.cell.2007.01.036 17382883

[4] Bozkurt G, Stjepanovic G, Vilardi F, Amlacher S, Wild K, Bange G, Favaloro V, Rippe K, Hurt E, Dobberstein B, Sinning I. Structural insights into tail-anchored protein binding and membrane insertion by Get3. Proc Natl Acad Sci U S A. 2009; 106(50):21131–6. doi:http://dx.doi.org/10.1073/pnas.0910223106. 10.1073/pnas.0910223106 PMC2795547 19948960

[5] Hu J, Li J, Qian X, Denic V, Sha B. The crystal structures of yeast Get3 suggest a mechanism for tail-anchored protein membrane insertion. PLoS ONE. 2009; 4(11):8061. doi:http://dx.doi.org/10.1371/journal.pone.0008061. 10.1371/journal.pone.0008061 PMC2778870 19956640

[6] Mateja A, Szlachcic A, Downing ME, Dobosz M, Mariappan M, Hegde RS, Keenan RJ. The structural basis of tail-anchored membrane protein recognition by Get3. Nature. 2009; 461(7262):361–6. doi:http://dx.doi.org/10.1038/nature08319. 10.1038/nature08319 PMC6528170 19675567

[7] Suloway CJM, Chartron JW, Zaslaver M, Clemons WM. Model for eukaryotic tail-anchored protein binding based on the structure of Get3. Proc Natl Acad Sci U S A. 2009; 106(35):14849–54. doi:http://dx.doi.org/10.1073/pnas.0907522106. 10.1073/pnas.0907522106 PMC2736419 19706470

[8] Yamagata A, Mimura H, Sato Y, Yamashita M, Yoshikawa A, Fukai S. Structural insight into the membrane insertion of tail-anchored proteins by Get3. Genes Cells Devoted Mol Cell Mech. 2010; 15(1):29–41. doi:http://dx.doi.org/10.1111/j.1365-2443.2009.01362.x. 10.1111/j.1365-2443.2009.01362.x 20015340

[9] Kutay U, Hartmann E, Rapoport TA (1993). A class of membrane proteins with a C-terminal anchor. Trends Cell Biol.

[10] Kutay U, Ahnert-Hilger G, Hartmann E, Wiedenmann B, Rapoport TA (1995). Transport route for synaptobrevin via a novel pathway of insertion into the endoplasmic reticulum membrane. EMBO J.

[11] Käll L, Krogh A, Sonnhammer ELL (2004). A combined transmembrane topology and signal peptide prediction method. J Mol Biol.

[12] Rose A, Lorenzen S, Goede A, Gruening B, Hildebrand PW (2009). RHYTHM–a server to predict the orientation of transmembrane helices in channels and membrane-coils. Nucleic Acids Res.

[13] Vasylenko T, Liou Y-F, Chen H-A, Charoenkwan P, Huang H-L, Ho S-Y (2015). SCMPSP: Prediction and characterization of photosynthetic proteins based on a scoring card method. BMC Bioinforma.

[14] Kalbfleisch T, Cambon A, Wattenberg BW. A bioinformatics approach to identifying tail-anchored proteins in the human genome. Traffic (Copenhagen, Denmark). 2007; 8(12):1687–94. doi:http://dx.doi.org/10.1111/j.1600-0854.2007.00661.x. 10.1111/j.1600-0854.2007.00661.x 17892534

[15] Emanuelsson O, Brunak S, von Heijne G, Nielsen H. Locating proteins in the cell using TargetP, SignalP and related tools. Nat Protoc. 2007; 2(4):953–71. doi:http://dx.doi.org/10.1038/nprot.2007.131. 10.1038/nprot.2007.131 17446895

[16] Krogh A, Larsson B, von Heijne G, Sonnhammer EL. Predicting transmembrane protein topology with a hidden Markov model: application to complete genomes. J Mol Biol. 2001; 305(3):567–80. doi:http://dx.doi.org/10.1006/jmbi.2000.4315. 10.1006/jmbi.2000.4315 11152613

[17] Petersen TN, Brunak S, von Heijne G, Nielsen H. SignalP 4.0: discriminating signal peptides from transmembrane regions. Nat Methods. 2011; 8(10):785–6. doi:http://dx.doi.org/10.1038/nmeth.1701. 10.1038/nmeth.1701 21959131

[18] Sonnhammer EL, von Heijne G, Krogh A. A hidden Markov model for predicting transmembrane helices in protein sequences. In: Proceedings of The Sixth International Conference on Intelligent Systems for Molecular Biology (ISMB). Bethesda: National Center for Biotechnology Information, NLM/NIH: 1998. p. 175–82. 9783223

[19] Dilcher M, Veith B, Chidambaram S, Hartmann E, Schmitt HD, Fischer von Mollard G. Use1p is a yeast SNARE protein required for retrograde traffic to the ER. EMBO J. 2003; 22(14):3664–74. doi:http://dx.doi.org/10.1093/emboj/cdg339. 10.1093/emboj/cdg339 PMC165609 12853481

[20] Beilharz T, Egan B, Silver PA, Hofmann K, Lithgow T. Bipartite signals mediate subcellular targeting of tail-anchored membrane proteins in Saccharomyces cerevisiae. J Biol Chem. 2003; 278(10):8219–23. doi:http://dx.doi.org/10.1074/jbc.M212725200. 10.1074/jbc.M212725200 12514182

[21] Wagner KM, Rüegg M, Niemann A, Suter U. Targeting and function of the mitochondrial fission factor GDAP1 are dependent on its tail-anchor. PLoS ONE. 2009; 4(4):5160. doi:http://dx.doi.org/10.1371/journal.pone.0005160. 10.1371/journal.pone.0005160 PMC2659752 19340293

[22] Burri L, Lithgow T (2004). A complete set of SNAREs in yeast. Traffic (Copenhagen, Denmark).

[23] Kriechbaumer V, Shaw R, Mukherjee J, Bowsher CG, Harrison A-M, Abell BM. Subcellular distribution of tail-anchored proteins in Arabidopsis. Traffic (Copenhagen, Denmark). 2009; 10(12):1753–64. doi:http://dx.doi.org/10.1111/j.1600-0854.2009.00991.x. 10.1111/j.1600-0854.2009.00991.x 19843281

[24] Cherry JM, Hong EL, Amundsen C, Balakrishnan R, Binkley G, Chan ET, Christie KR, Costanzo MC, Dwight SS, Engel SR, Fisk DG, Hirschman JE, Hitz BC, Karra K, Krieger CJ, Miyasato SR, Nash RS, Park J, Skrzypek MS, Simison M, Weng S, Wong ED. Saccharomyces Genome Database: the genomics resource of budding yeast. Nucleic Acids Res. 2012; 40(Database issue):700–5. doi:http://dx.doi.org/10.1093/nar/gkr1029. 10.1093/nar/gkr1029 PMC3245034 22110037

[25] Lamesch P, Berardini TZ, Li D, Swarbreck D, Wilks C, Sasidharan R, Muller R, Dreher K, Alexander DL, Garcia-Hernandez M, Karthikeyan AS, Lee CH, Nelson WD, Ploetz L, Singh S, Wensel A, Huala E. The Arabidopsis Information Resource (TAIR): improved gene annotation and new tools. Nucleic Acids Res. 2012; 40(Database issue):1202–10. doi:http://dx.doi.org/10.1093/nar/gkr1090. 10.1093/nar/gkr1090 PMC3245047 22140109

[26] Tanz SK, Castleden I, Hooper CM, Vacher M, Small I, Millar HA. SUBA3: a database for integrating experimentation and prediction to define the SUBcellular location of proteins in Arabidopsis. Nucleic Acids Res. 2013; 41(Database issue):1185–91. doi:http://dx.doi.org/10.1093/nar/gks1151. 10.1093/nar/gks1151 PMC3531127 23180787

[27] Altschul SF, Madden TL, Schäffer AA, Zhang J, Zhang Z, Miller W, Lipman DJ (1997). Gapped BLAST and PSI-BLAST: a new generation of protein database search programs. Nucleic Acids Res.

[28] Sharpe HJ, Stevens TJ, Munro S. A comprehensive comparison of transmembrane domains reveals organelle-specific properties. Cell. 2010; 142(1):158–69. doi:http://dx.doi.org/10.1016/j.cell.2010.05.037. 10.1016/j.cell.2010.05.037 PMC2928124 20603021

[29] Carrondo MA, Spadon P (2011). Macromolecular Crystallography: Deciphering the Structure, Function and Dynamics of Biological Molecules. NATO Science for Peace and Security Series A: Chemistry and Biology.

[30] Kahsay RY, Gao G, Liao L. An improved hidden Markov model for transmembrane protein detection and topology prediction and its applications to complete genomes. Bioinformatics. 2005; 21(9):1853–8. doi:http://dx.doi.org/10.1093/bioinformatics/bti303. 10.1093/bioinformatics/bti303 15691854

[31] Viterbi A (2006). Error Bounds for Convolutional Codes and an Asymptotically Optimum Decoding Algorithm. IEEE Trans Inf Theor.

