# Supplementary material for: Development of a prediction system for tail-anchored proteins
Source: BMC Bioinformatics. 2016 Sep 15;17:378. doi: 10.1186/s12859-016-1202-7 (PMC5025589; doi:10.1186/s12859-016-1202-7)
Supplement: Additional file 1 — Table S5. Sequences that were not predicted. (PDF 40 kb) [file 12859_2016_1202_MOESM1_ESM.pdf]

Table S2. Initial conditional probability in the TA model

Amino acid	Output probability			
	Tail region	TMD region	Cap region	Globular region
A	0.0273	0.1333	0.01	0.05
C	0.0778	0.0143	0.01	0.05
D	0.0778	0.0143	0.01	0.05
E	0.0778	0.0143	0.01	0.05
F	0.0273	0.1333	0.01	0.05
G	0.0273	0.1333	0.01	0.05
H	0.0778	0.0143	0.01	0.05
I	0.0273	0.1333	0.01	0.05
K	0.0778	0.0143	0.41	0.05
L	0.0273	0.1333	0.01	0.05
M	0.0273	0.0143	0.01	0.05
N	0.0778	0.0143	0.01	0.05
P	0.0273	0.0143	0.01	0.05
Q	0.0778	0.0143	0.01	0.05
R	0.0778	0.0143	0.41	0.05
S	0.0778	0.0143	0.01	0.05
T	0.0778	0.0143	0.01	0.05
V	0.0273	0.1333	0.01	0.05
W	0.0273	0.0143	0.01	0.05
Y	0.0778	0.0143	0.01	0.05

Output probabilities were set such that hydrophilic, hydrophobic, and basic residues were more likely to occur in the tail region, TMD region, and cap region, respectively.
