# Supplementary figures and images for: Development of a prediction system for tail-anchored proteins
Source: BMC Bioinformatics. 2016 Sep 15;17:378. doi: 10.1186/s12859-016-1202-7 (PMC5025589; doi:10.1186/s12859-016-1202-7)

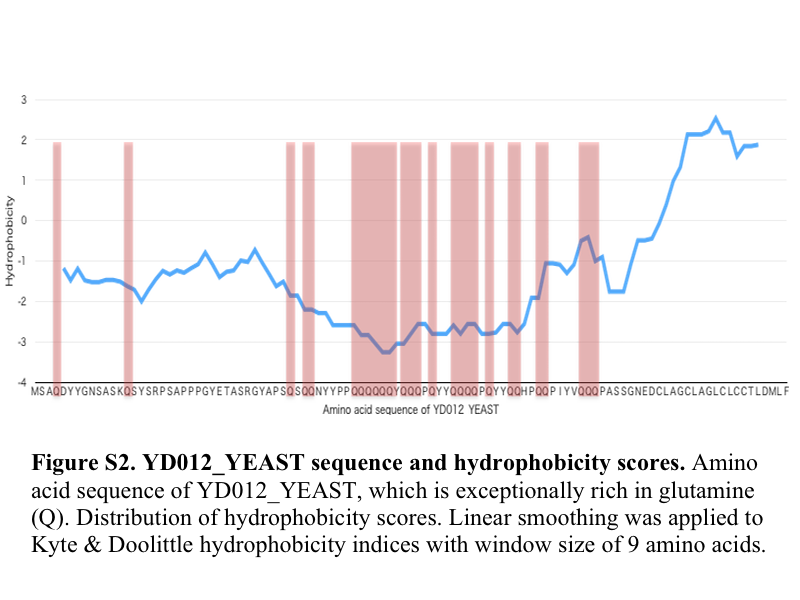

Supplement: Additional file 2 — Figure S2. YD012_YEAST sequence and hydrophobicity scores. (A): Amino acid sequence of YD012_YEAST, which is exceptionally rich in glutamine (Q). (B): Distribution of hydrophobicity scores. Linear smoothing was applied to the Kyte-Doolittle hydrophobicity indices. (TIF 162 kb) [file 12859_2016_1202_MOESM2_ESM.tif]

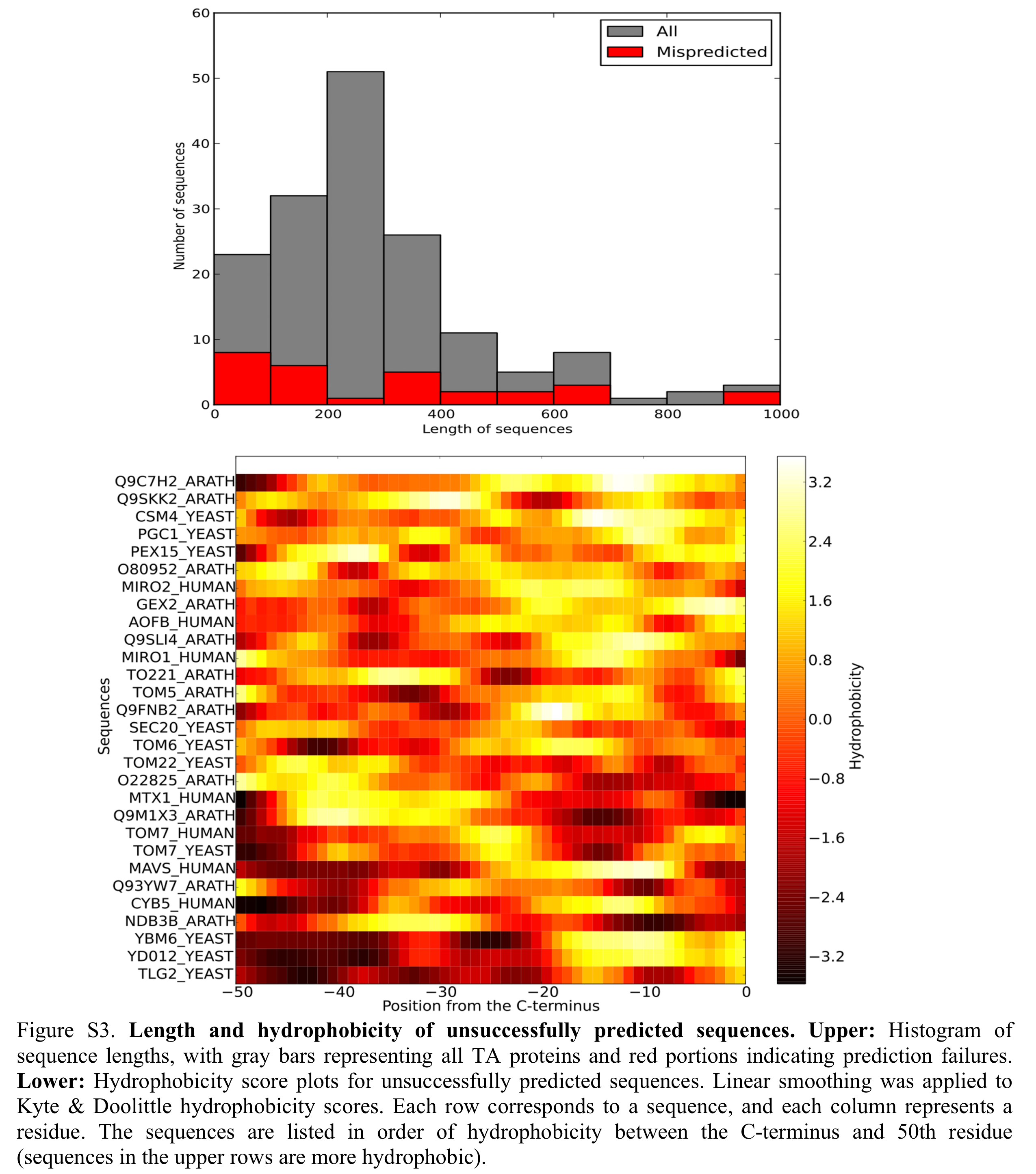

Supplement: Additional file 3 — Figure S3. Lengths and hydrophobicities of sequences that were not predicted. Upper: Sequence lengths. The gray bars represent all TA proteins and red portions indicating prediction failures. Lower: Hydrophobicity scores of unsuccessfully predicted sequences. Linear smoothing was applied to Kyte–Doolittle hydrophobicity scores. Each row corresponds to a sequence, and each column represents a residue. The sequences are listed in order of hydrophobicity between the C-terminus and the 50th residue (sequences in the upper rows are more hydrophobic). (TIF 1658 kb) [file 12859_2016_1202_MOESM3_ESM.tif]

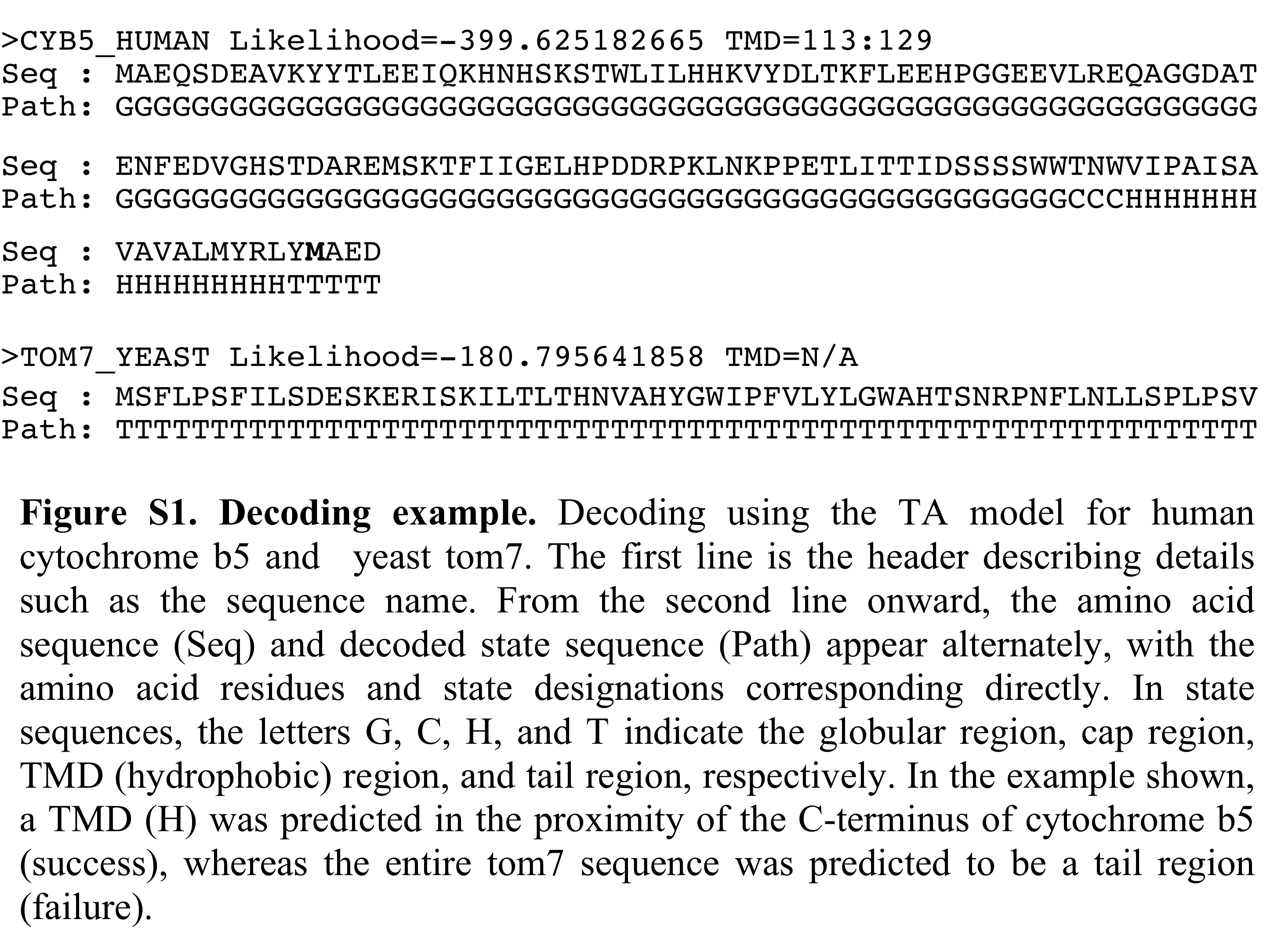

Supplement: Additional file 8 — Figure S1. Decoding example. Decoding using the TA model for human cytochrome b5 and yeast TOM7. The first line is the header describing details such as the sequence name. From the second line onward, the amino acid sequence (Seq) and decoded state sequence (Path) appear alternately with direct correspondence between the amino acid residues and state designations. In state sequences, the letters G, C, H, and T indicate the globular, cap, TMD (hydrophobic), and tail regions, respectively. In the example shown, a TMD (H) was predicted in the proximity of the C-terminus of cytochrome b5 (success), whereas the entire TOM7 sequence was predicted to be a tail region (failure). (TIF 864 kb) [file 12859_2016_1202_MOESM8_ESM.tif]
