# Supplementary material for: Development of a prediction system for tail-anchored proteins
Source: BMC Bioinformatics. 2016 Sep 15;17:378. doi: 10.1186/s12859-016-1202-7 (PMC5025589; doi:10.1186/s12859-016-1202-7)
Supplement: Additional file 4 — Table S1. Empirically validated TA proteins. (PDF 119 kb) [file 12859_2016_1202_MOESM4_ESM.pdf]

Table S1. Empirically substantiated TA proteins

UniProt ID	Subcellular Location	Method	References
AOFB_HUMAN	Mitochondria	immunofluorescence	[1]
SYJ2B_HUMAN	Mitochondria	immunofluorescence	[2]
CYB5B_HUMAN	ER	fractionation	[3]
BAX_HUMAN	mitochondria	fluorescence	[4, 5, 6, 7, 8]
MIRO1_HUMAN	mitochondria	fluorescence	[9, 10]
MIRO2_HUMAN	mitochondria	fluorescence	[9, 11]
FIS1_HUMAN	mitochondria	immunofluorescence, fluorescence	[12, 13, 14, 15]
MAVS_HUMAN	mitochondria, peroxisome	immunofluorescence (maybe)	[16, 17]
GDAP1_HUMAN	mitochondria	fluorescence, immunofluorescence	[18]
MTX1_HUMAN	mitochondria	similarity (fluorescence; GFP)	[19, 20]
TOM7_HUMAN	mitochondria	fluorescence	[21]
CYB5_HUMAN	mitochondria	immunofluorescence	[22]
HMOX1_HUMAN	ER	fluorescence (CFP)	[23]
SC61B_HUMAN	ER	fractionation	[24]
SC61G_HUMAN	ER		[24]
AL3A2_HUMAN	ER (microsome)	fractionation	[25]
PTN1_HUMAN	ER	fluorescence	[26]
VAPA_HUMAN	ER, Golgi, plasma, nucleus	immunofluorescence, fractionation	[27, 28]
SLMAP_HUMAN	plasma membrane	immunofluorescence	[29]
STX1A_HUMAN	synaptic vesicle membrane	similarity (immunofluorescence)	[30]
STX1B_HUMAN	plasma membrane	immunofluorescence	[31]
STX2_HUMAN	plasma membrane	fractionation, fluorescence	[30, 32, 33]
STX3_HUMAN	plasma membrane	fractionation, fluorescence	[32, 33]
STX5_HUMAN	ER, Golgi	fluorescence	[30, 34, 35]
STX4_HUMAN	plasma membrane	fractionation, fluorescence	[30, 32, 33]
STX6_HUMAN	Golgi	immunofluorescence	[35, 36]
STX7_HUMAN	Golgi	fluorescence	[35, 37]
STX8_HUMAN	ER, plasma	antibody	[35, 38]
STX10_HUMAN	Golgi	immunofluorescence	[39]
STX12_HUMAN	Golgi	solubilize with detergent, immunofluorescence	[40]
STX16_HUMAN	Golgi	immunofluorescence	[41]
STX18_HUMAN	ER	fractionation, immunofluorescence	[42, 43]
GOSR1_HUMAN	Golgi	fractionation, immunofluorescence	[34, 44, 45, 46, 47]
BET1_HUMAN	Golgi	similarity (immunofluorescence)	[48]
VAMP1_HUMAN	synaptic vesicle membrane, mitochondria	immunofluorescence	[49, 50]
VAMP2_HUMAN	synaptic vesicle membrane, plasma membrane	immunofluorescence	[50]
VAMP3_HUMAN	endosome	immunofluorescence	[51]
VAMP4_HUMAN	Golgi, lysosome, endosome	immunofluorescence	[52]
VAMP5_HUMAN	plasma membrane	immunofluorescence	[53]
VAMP8_HUMAN	plasma membrane	immunofluorescence	[52, 54]
VAMP7_HUMAN	ER	immunofluorescence, immunoelectroscopy	[52, 55]
FMP32_YEAST	mitochondria	GFP, ms, fractionation	[56, 57, 58, 59]
FIS1_YEAST	mitochondria	GFP, fractionation	[56, 59, 60]
TOM5_YEAST	mitochondria	direct assay, fractionation, ms	[58, 59, 61]
TOM6_YEAST	mitochondria	fractionation, complex	[58, 59]
TOM7_YEAST	mitochondria	direct assay	[58, 59, 62]

Continued on next page

TOM22_YEAST	mitochondria	fractionation, ms	[58, 59, 63]
PRM3_YEAST	nuclear outer membrane	GFP	[56, 64, 65, 66, 67]
KAR1_YEAST	nuclear outer membrane	inference, GFP	[68, 69]
UBC6_YEAST	ER, plasma (possibly)	immunofluorescence	[70]
SCS2_YEAST	ER, nuclear	immunofluorescence, fluorescence	[57, 71, 72, 73]
CSM4_YEAST	ER, nuclear	fluorescence	[56]
HLJ1_YEAST	ER	fluorescence	[56]
YSY6_YEAST	ER	fluorescence	[57, 74]
SC6B2_YEAST	ER	inference (part of a complex)	[75]
DPM1_YEAST	ER	fluorescence,	[57, 76]
SC61G_YEAST	ER	fluorescence, part of a known complex	[57]
SC6B1_YEAST	ER	inference (part of a complex)	[77]
PGC1_YEAST	mitochondria, lipid particles	fluorescence, fractionation	[56, 58, 59, 78]
PEX15_YEAST	ER, peroxisome	fluorescence	[57, 79]
CYB5_YEAST	ER	immunofluorescence	[80, 81]
HPH1_YEAST	ER	fluorescence	[56, 82]
HPH2_YEAST	ER	fluorescence	[82]
FAR10_YEAST	ER	fluorescence	[56]
VPS64_YEAST	ER	fluorescence	[56, 57]
SEC20_YEAST	ER	treatment with sodium carbonate	[83]
SEC22_YEAST	ER, Golgi	immunofluorescence	[84, 85]
UFE1_YEAST	ER	immunofluorescence	[86]
USE1_YEAST	ER	immunofluorescence, fluorescence, sodium carbonate	[57, 87, 88]
SED5_YEAST	Golgi	fluorescence, immunofluorescence, sodium carbonate	[89, 90]
GOSR1_YEAST	Golgi	immunofluorescence	[91, 92]
BET1_YEAST	ER, possibly Golgi	fractionation	[93]
BOS1_YEAST	ER, possibly Golgi	fractionation	[93]
TLG1_YEAST	trans-Golgi network (TGN)	immunofluorescence, fractionation, solubilization	[94, 95]
SFT1_YEAST	Golgi	immunofluorescence	[96]
TLG2_YEAST	trans-Golgi network (TGN)	immunofluorescence, fluorescence	[95, 97]
STX8_YEAST	endosome, Golgi	fluorescence	[98]
PEP12_YEAST	Golgi	fractionation, solubilization	[99]
VTI1_YEAST	Golgi	immunofluorescence, solubilization	[100]
NYV1_YEAST	vacuole	fluorescence	[57]
VAM3_YEAST	vacuole	immunofluorescence	[101]
SNC1_YEAST	vacuole, plasma, Golgi	fluorescence	[102, 103]
SNC2_YEAST	Golgi	fluorescence	[103, 104]
SSO1_YEAST	plasma, vacuole	fluorescence	[105]
SSO2_YEAST	plasma	fluorescence	[105]
YD012_YEAST	plasma	fluorescence	[56, 57]
YBM6_YEAST	plasma	fluorescence	[56]
PHM6_YEAST	vacuole	fluorescence	[57]
Q9SMS0_ARATH	chloroplast	large-scale analysis	[106]
Q9FJT9_ARATH	chloroplast, mitochondria, plastid	large-scale analysis	[106, 107]
Q9M1X3_ARATH	thylakoid, chloroplast envelope, chloroplast,	large-scale analysis, fractionation	[106, 108, 109, 110, 111, 112]
Q9SKK2_ARATH	chloroplast	large-scale analysis	[112]
OEP61_ARATH	plastid	fluorescence, solubilization (Na2CO3),	[113, 114]
Q9FDW8_ARATH	plastid	fluorescence, immunofluorescence,	[115]

Continued on next page

Q9SLI4_ARATH	chloroplast, thylakoid membrane	large-scale analysis	[106, 108, 111]
Q8S8K0_ARATH	chloroplast, thylakoid membrane	large-scale analysis	[106, 111]
O80952_ARATH	chloroplast envelope, mitochondria	large-scale analysis, fluorescence	[110, 116, 117]
TOM5_ARATH	mitochondria, plastid	large-scale analysis	[107, 118]
Q94K78_ARATH	mitochondria	large-scale analysis	[119, 120, 121]
TO221_ARATH	mitochondria	complex	[122]
TO202_ARATH	mitochondria	large-scale analysis, part of a known complex	[118, 119, 123]
TO201_ARATH	mitochondria	complex	[123]
Q944S8_ARATH	mitochondria, vacuole membrane	large-scale analysis	[119, 124]
Q9LXR8_ARATH	mitochondria, peroxisome	fluorescence, large-scale analysis	[118, 119, 125]
Q9SIQ8_ARATH	mitochondria	fractionation, solubilization, large-scale analysis	[119, 126]
TO203_ARATH	mitochondria	fractionation (ms), complex	[123, 127]
O64471_ARATH	mitochondria, plastid*	fractionation (ms), large-scale analysis	[107, 118, 119, 127]
TO222_ARATH	mitochondria, vacuole membrane*	fractionation (ms), large-scale analysis	[107, 118, 123, 127]
NDB3B_ARATH	mitochondria	extraction (Na_2CO_3), fractionation	[126]
TO204_ARATH	mitochondria, vacuole membrane*	extraction (Na_2CO_3), fractionation, complex, large-scale analysis	[123, 124, 126]
Q9SHJ6_ARATH	mitochondria	fluorescence (YFP, GFP), fractionation, large-scale analysis	[107, 118, 124, 125, 128]
NDB3A_ARATH	mitochondria	fractionation (ms), large-scale analysis, complex	[120, 129]
Q8RXF8_ARATH	mitochondria	fluorescence, large-scale analysis	[119, 120, 130]
TOM6_ARATH	mitochondria	fractionation (ms), large-scale analysis	[127, 131]
Q9C7G5_ARATH	mitochondria	fractionation (ms)	[132]
O22825_ARATH	mitochondria	fractionation (ms), large-scale analysis	[118]
Q93YW7_ARATH	mitochondria	fluorescence	[133]
Q9FNB2_ARATH	mitochondria	large-scale analysis	[107, 119]
Q9C7H2_ARATH	ER	fractionation (ms), large-scale analysis	[118, 134]
O23037_ARATH	ER	fluorescence	[135, 136]
Q9ZWT2_ARATH	ER	fluorescence	[115, 137]
STLP2_ARATH	ER, plasma	fractionation, solubilization, fluorescence (GFP, YFP), large-scale analysis	[107, 138, 139]
SYP61_ARATH	Golgi	fluorescence	[140, 141]
SYP72_ARATH	ER	fluorescence	[141]
CYB51_ARATH	ER, plasma	fluorescence, large-scale analysis	[107, 137]
SYP71_ARATH	ER, plasma	fluorescence (YFP, GFP), large-scale analysis	[107, 141, 142]
FDFT_ARATH	ER, plasma	fluorescence, large-scale analysis	[107, 134, 143]
SYP73_ARATH	ER	fluorescence	[141, 144]
VAP22_ARATH	plasma	large-scale analysis	[134, 145]
CYB52_ARATH	ER	fluorescence, large-scale analysis	[115, 118, 137, 146]
O65688_ARATH	ER	fluorescence	[143]
SEC22_ARATH	ER, Golgi	fluorescence (GFP, CFP), large-scale analysis	[145, 147, 148, 149, 150, 151]
VTI11_ARATH	vacuole, Golgi	fluorescence	[141, 152, 153, 154]
SYP81_ARATH	ER	fluorescence	[141, 149]
VAP12_ARATH	ER, plasma	fluorescence, large-scale analysis	[145, 151, 155]
VAP11_ARATH	plasma, ER	immunofluorescence, large-scale analysis	[107, 145, 156, 157]

Continued on next page

**S1 table** – *Continued from previous page*			
O23465_ARATH	ER, plasma	solubilization, large-scale analysis	[134, 158]
O23144_ARATH	ER, plasma	fractionation, fluorescence	[159, 160]
Q9ZVC6_ARATH	ER	fluorescence, large-scale analysis	[134, 161]
GOS12_ARATH	vacuole, Golgi	fluorescence, large-scale analysis	[134, 141, 162]
VTI12_ARATH	plasma	fluorescence, immunofluorescence	[141, 152, 163]
GOS11_ARATH	Golgi	fluorescence, large-scale analysis	[141, 164]
MEM12_ARATH	Golgi	fluorescence	[141]
SYP31_ARATH	Golgi	fluorescence, immunofluorescence	[141, 165]
MEM11_ARATH	Golgi	fluorescence (GFP, YFP)	[141, 166, 167]
BET11_ARATH	Golgi, plasma	fluorescence, large-scale analysis	[141, 145, 167]
SYP32_ARATH	Golgi	fluorescence, large-scale analysis	[134, 141]
SY124_ARATH	plasma	fluorescence, large-scale analysis	[134, 141, 145, 151, 168, 169]
SY132_ARATH	plasma	fluorescence, large-scale analysis	[134, 141, 145, 148, 151, 168, 170]
Q8S8J6_ARATH	plasma	large-scale analysis	[134, 151, 158]
SY125_ARATH	plasma	fluorescence, large-scale analysis	[141, 168]
SY131_ARATH	plasma	fluorescence, large-scale analysis	[141, 145, 168]
S61G3_ARATH	plasma	large-scale analysis	[145]
VA726_ARATH	plastid, plasma	fluorescence (GFP), large-scale analysis	[112, 141, 171]
NPS12_ARATH	plasma	fluorescence, large-scale analysis	[141, 145, 151]
FKB42_ARATH	ER	fluorescence, immunofluorescence	[172, 173, 174, 175]
Q944S5_ARATH	plasma	large-scale analysis	[176]
VAP22_ARATH	plasma	large-scale analysis	[134, 145, 176]
SY111_ARATH	plasma	fluorescence, large-scale analysis	[141, 145, 168, 177]
GEX2_ARATH	plasma	fluorescence	[178]
VAP21_ARATH	plasma, ER	large-scale analysis	[118, 176]
NPS11_ARATH	plasma	fluorescence, large-scale analysis	[141, 176]
Q9SN26_ARATH	plasma	large-scale analysis	[176]
SY123_ARATH	plasma	fluorescence	[141, 168]
Q9SRZ3_ARATH	plasma	large-scale analysis	[176]
PAS1_ARATH	nucleus, cytosol, plasma	fluorescence, large-scale analysis	[134, 179, 180, 181]
SY112_ARATH	plasma, nucleus	ms, GFP	[141, 182]
NAC78_ARATH	nucleus	fluorescence	[183]

Fluorescence refers to any method that detects subcellular localization by binding of GFP or YFP to the target sequence. Immunofluorescence is a method involving sequences marked with histidine tags or other markers and antibodies to which fluorescent proteins such as GFP are bound. Large-scale analysis refers to a thorough investigation of sequences within a given organelle fraction using tandem mass spectrometry (MS/MS) and other similar techniques. Extraction (Na_2CO_3) indicates a method to confirm the presence or absence of a TMD by sodium carbonate treatment.

References

[1] Long GL, Winfield S, Adolph KW, Ginns EI, Bornstein P. Structure and organization of the human metaxin gene (MTX) and pseudogene. Genomics. 1996;33(2):177–84.

[2] Chehab EW, Patharkar OR, Cushman JC. Isolation and characterization of a novel v-SNARE family protein that interacts with a calcium-dependent protein kinase from the common ice plant, Mesembryanthemum crystallinum. Planta. 2007;225(4):783–99.

[3] Geisler M, Kolukisaoglu HU, Bouchard R, Billion K, Berger J, Saal B, et al. TWISTED DWARF1, a unique plasma membrane-anchored immunophilin-like protein, interacts with Arabidopsis multidrug resistance-like transporters AtPGP1 and AtPGP19. Molecular biology of the cell. 2003;14(10):4238–49.

[4] Uemura T, Sato MH, Takeyasu K. The longin domain regulates subcellular targeting of VAMP7 in Arabidopsis thaliana. FEBS letters. 2005;579(13):2842–6.

[5] Heazlewood JL, Tonti-Filippini JS, Gout AM, Day DA, Whelan J, Millar AH. Experimental analysis of the Arabidopsis mitochondrial proteome highlights signaling and regulatory components, provides assessment of targeting prediction programs, and indicates plant-specific mitochondrial proteins. The Plant cell. 2004;16(1):241–56.

[6] Smyczynski C, Roudier F, Gissot L, Vaillant E, Grandjean O, Morin H, et al. The C terminus of the immunophilin PASTICCINO1 is required for plant development and for interaction with a NAC-like transcription factor. The Journal of biological chemistry. 2006;281(35):25475–84.

[7] Enami K, Ichikawa M, Uemura T, Kutsuna N, Hasezawa S, Nakagawa T, et al. Differential expression control and polarized distribution of plasma membrane-resident SYP1 SNAREs in Arabidopsis thaliana. Plant & cell physiology. 2009;50(2):280–9.

[8] Hirose H, Arasaki K, Dohmae N, Takio K, Hatsuzawa K, Nagahama M, et al. Implication of ZW10 in membrane trafficking between the endoplasmic reticulum and Golgi. The EMBO journal. 2004;23(6):1267–78.

[9] Brugière S, Kowalski S, Ferro M, Seigneurin-Berny D, Miras S, Salvi D, et al. The hydrophobic proteome of mitochondrial membranes from Arabidopsis cell suspensions. Phytochemistry. 2004;65(12):1693–707.

[10] Kamphausen T, Fanghänel J, Neumann D, Schulz B, Rahfeld JU. Characterization of Arabidopsis thaliana AtFKBP42 that is membrane-bound and interacts with Hsp90. The Plant journal : for cell and molecular biology. 2002;32(3):263–76.

[11] Meyer EH, Taylor NL, Millar AH. Resolving and identifying protein components of plant mitochondrial respiratory complexes using three dimensions of gel electrophoresis. Journal of proteome research. 2008;7(2):786–94.

[12] DaSilva LLP, Snapp EL, Denecke J, Lippincott-Schwartz J, Hawes C, Brandizzi F. Endoplasmic reticulum export sites and Golgi bodies behave as single mobile secretory units in plant cells. The Plant cell. 2004;16(7):1753–71.

[13] Sweet DJ, Pelham HR. The Saccharomyces cerevisiae SEC20 gene encodes a membrane glycoprotein which is sorted by the HDEL retrieval system. The EMBO journal. 1992;11(2):423–32.

[14] Halbach A, Landgraf C, Lorenzen S, Rosenkranz K, Volkmer-Engert R, Erdmann R, et al. Targeting of the tail-anchored peroxisomal membrane proteins PEX26 and PEX15 occurs through C-terminal PEX19-binding sites. Journal of cell science. 2006;119(Pt 12):2508–17.

[15] Ossig R, Dascher C, Trepte HH, Schmitt HD, Gallwitz D. The yeast SLY gene products, suppressors of defects in the essential GTP-binding Ypt1 protein, may act in endoplasmic reticulum-to-Golgi transport. Molecular and cellular biology. 1991;11(6):2980–93.

[16] Tartakoff AM, Jaiswal P. Nuclear fusion and genome encounter during yeast zygote formation. Molecular biology of the cell. 2009;20(12):2932–42.

[17] Macasev D. How Do Plant Mitochondria Avoid Importing Chloroplast Proteins? Components of the Import Apparatus Tom20 and Tom22 from Arabidopsis Differ from Their Fungal Counterparts. PLANT PHYSIOLOGY. 2000;123(3):811–816.

[18] Carter C, Pan S, Zouhar J, Avila EL, Girke T, Raikhel NV. The vegetative vacuole proteome of Arabidopsis thaliana reveals predicted and unexpected proteins. The Plant cell. 2004;16(12):3285–303.

[19] Sunderhaus S, Dudkina NV, Jänsch L, Klodmann J, Heinemeyer J, Perales M, et al. Carbonic anhydrase subunits form a matrix-exposed domain attached to the membrane arm of mitochondrial complex I in plants. The Journal of biological chemistry. 2006;281(10):6482–8.

[20] Tai G, Lu L, Wang TL, Tang BL, Goud B, Johannes L, et al. Participation of the syntaxin 5/Ykt6/GS28/GS15 SNARE complex in transport from the early/recycling endosome to the trans-Golgi network. Molecular biology of the cell. 2004;15(9):4011–22.

[21] McNew JA, Coe JGS, Sø gaard M, Zemelman BV, Wimmer C, Hong W, et al. Gos1p, a Saccharomyces cerevisiae SNARE protein involved in Golgi transport. FEBS Letters. 1998;435(1):89–95.

[22] Yamaoka S, Leaver CJ. EMB2473/MIRO1, an Arabidopsis Miro GTPase, is required for embryogenesis and influences mitochondrial morphology in pollen. The Plant cell. 2008;20(3):589–601.

[23] Faulhammer F, Konrad G, Brankatschk B, Tahirovic S, Knödler A, Mayinger P. Cell growth-dependent coordination of lipid signaling and glycosylation is mediated by interactions between Sac1p and Dpm1p. The Journal of cell biology. 2005;168(2):185–91.

[24] Huh WK, Falvo JV, Gerke LC, Carroll AS, Howson RW, Weissman JS, et al. Global analysis of protein localization in budding yeast. Nature. 2003;425(6959):686–91.

[25] Wooding S, Pelham HR. The dynamics of golgi protein traffic visualized in living yeast cells. Molecular biology of the cell. 1998;9(9):2667–80.

[26] Sanderfoot AA. The Arabidopsis Genome. An Abundance of Soluble N-Ethylmaleimide-Sensitive Factor Adaptor Protein Receptors. PLANT PHYSIOLOGY. 2000;124(4):1558–1569.

[27] Pedrazzini E. Mechanism of Residence of Cytochrome b(5), a Tail-anchored Protein, in the Endoplasmic Reticulum. The Journal of Cell Biology. 2000;148(5):899–914.

[28] Brickner JH, Walter P. Gene recruitment of the activated INO1 locus to the nuclear membrane. PLoS biology. 2004;2(11):e342.

[29] Koh S, André A, Edwards H, Ehrhardt D, Somerville S. Arabidopsis thaliana subcellular responses to compatible Erysiphe cichoracearum infections. The Plant journal : for cell and molecular biology. 2005;44(3):516–29.

[30] Hönlinger a, Bömer U, Alconada a, Eckerskorn C, Lottspeich F, Dietmeier K, et al. Tom7 modulates the dynamics of the mitochondrial outer membrane translocase and plays a pathway-related role in protein import. The EMBO journal. 1996;15(9):2125–37.

[31] Komiya T. Metaxin Is a Component of a Preprotein Import Complex in the Outer Membrane of the Mammalian Mitochondrion. Journal of Biological Chemistry. 1997;272(10):6510–6518.

[32] Kasai K, Akagawa K. Roles of the cytoplasmic and transmembrane domains of syntaxins in intracellular localization and trafficking. Journal of cell science. 2001;114(Pt 17):3115–24.

[33] Kleffmann T, Russenberger D, von Zychlinski A, Christopher W, Sjölander K, Gruissem W, et al. The Arabidopsis thaliana chloroplast proteome reveals pathway abundance and novel protein functions. Current biology : CB. 2004;14(5):354–62.

[34] Zybailov B, Rutschow H, Friso G, Rudella A, Emanuelsson O, Sun Q, et al. Sorting signals, N-terminal modifications and abundance of the chloroplast proteome. PloS one. 2008;3(4):e1994.

[35] Schuldiner M, Metz J, Schmid V, Denic V, Rakwalska M, Schmitt HD, et al. The GET complex mediates insertion of tail-anchored proteins into the ER membrane. Cell. 2008;134(4):634–45.

[36] Newman AP, Groesch ME, Ferro-Novick S. Bos1p, a membrane protein required for ER to Golgi transport in yeast, co-purifies with the carrier vesicles and with Bet1p and the ER membrane. The EMBO journal. 1992;11(10):3609–17.

[37] Lewis MJ, Pelham HRB. SNARE-Mediated Retrograde Traffic from the Golgi Complex to the Endoplasmic Reticulum. Cell. 1996;85(2):205–215.

[38] Bernstein AM, Whiteheart SW. Identification of a Cellubrevin/Vesicle Associated Membrane Protein 3Homologue in Human Platelets. Blood. 1999;93(2):571–579.

[39] Sommer T, Jentsch S. A protein translocation defect linked to ubiquitin conjugation at the endoplasmic reticulum. Nature. 1993;365(6442):176–9.

[40] Bonza MC, Fusca T, Homann U, Thiel G, De Michelis MI. Intracellular localisation of PPI1 (proton pump interactor, isoform 1), a regulatory protein of the plasma membrane H(+)-ATPase of Arabidopsis thaliana. Plant biology (Stuttgart, Germany). 2009;11(6):869–77.

[41] Hay JC. Localization, Dynamics, and Protein Interactions Reveal Distinct Roles for ER and Golgi SNAREs. The Journal of Cell Biology. 1998;141(7):1489–1502.

[42] Werhahn W, Jänsch L, Braun HP. Identification of novel subunits of the TOM complex from Arabidopsis thaliana. Plant Physiology and Biochemistry. 2003;41(5):407–416.

[43] Maggio C, Barbante A, Ferro F, Frigerio L, Pedrazzini E. Intracellular sorting of the tail-anchored protein cytochrome b5 in plants: a comparative study using different isoforms from rabbit and Arabidopsis. Journal of experimental botany. 2007;58(6):1365–79.

[44] Morishita T, Kojima Y, Maruta T, Nishizawa-Yokoi A, Yabuta Y, Shigeoka S. Arabidopsis NAC transcription factor, ANAC078, regulates flavonoid biosynthesis under high-light. Plant & cell physiology. 2009;50(12):2210–22.

[45] Jaquinod M, Villiers F, Kieffer-Jaquinod S, Hugouvieux V, Bruley C, Garin J, et al. A proteomics dissection of Arabidopsis thaliana vacuoles isolated from cell culture. Molecular & cellular proteomics : MCP. 2007;6(3):394–412.

[46] Schweiger R, Müller NC, Schmitt MJ, Soll J, Schwenkert S. AtTPR7 is a chaperone docking protein of the Sec translocon in Arabidopsis. Journal of cell science. 2012;.

[47] Lupashin VV, Pokrovskaya ID, McNew JA, Waters MG. Characterization of a novel yeast SNARE protein implicated in Golgi retrograde traffic. Molecular biology of the cell. 1997;8(12):2659–76.

[48] Sickmann A, Reinders J, Wagner Y, Joppich C, Zahedi R, Meyer HE, et al. The proteome of Saccharomyces cerevisiae mitochondria. Proceedings of the National Academy of Sciences of the United States of America. 2003;100(23):13207–12.

[49] Wu G, Otegui MS, Spalding EP. The ER-localized TWD1 immunophilin is necessary for localization of multidrug resistance-like proteins required for polar auxin transport in Arabidopsis roots. The Plant cell. 2010;22(10):3295–304.

[50] Zhao X, Jäntti J. Functional characterization of the trans-membrane domain interactions of the Sec61 protein translocation complex beta-subunit. BMC cell biology. 2009;10(1):76.

[51] Mullock BM, Smith CW, Ihrke G, Bright NA, Lindsay M, Parkinson EJ, et al. Syntaxin 7 Is Localized to Late Endosome Compartments, Associates with Vamp 8, and Is Required for Late Endosome-Lysosome Fusion. Mol Biol Cell. 2000;11(9):3137–3153.

[52] Babiychuk E, Müller F, Eubel H, Braun HP, Frentzen M, Kushnir S. Arabidopsis phosphatidylglycerophosphate synthase 1 is essential for chloroplast differentiation, but is dispensable for mitochondrial function. The Plant Journal. 2003;33(5):899–909.

[53] Xu Y, Zhang F, Su Z, McNew JA, Shin YK. Hemifusion in SNARE-mediated membrane fusion. Nature structural & molecular biology. 2005;12(5):417–22.

[54] Drakakaki G, van de Ven W, Pan S, Miao Y, Wang J, Keinath NF, et al. Isolation and proteomic analysis of the SYP61 compartment reveal its role in exocytic trafficking in Arabidopsis. Cell research. 2012;22(2):413–24.

[55] Simocková M, Holic R, Tahotná D, Patton-Vogt J, Griac P. Yeast Pgc1p (YPL206c) controls the amount of phosphatidylglycerol via a phospholipase C-type degradation mechanism. The Journal of biological chemistry. 2008;283(25):17107–15.

[56] Marmagne A, Ferro M, Meinnel T, Bruley C, Kuhn L, Garin J, et al. A high content in lipid-modified peripheral proteins and integral receptor kinases features in the arabidopsis plasma membrane proteome. Molecular & cellular proteomics : MCP. 2007;6(11):1980–96.

[57] Uemura T, Ueda T, Ohniwa RL, Nakano A, Takeyasu K, Sato MH. Systematic analysis of SNARE molecules in Arabidopsis: dissection of the post-Golgi network in plant cells. Cell structure and function. 2004;29(2):49–65.

[58] Hattier T, Andrulis ED, Tartakoff AM. Immobility, inheritance and plasticity of shape of the yeast nucleus. BMC cell biology. 2007;8(1):47.

[59] Aung K, Hu J. The Arabidopsis tail-anchored protein PEROXISOMAL AND MITOCHONDRIAL DIVISION FACTOR1 is involved in the morphogenesis and proliferation of peroxisomes and mitochondria. The Plant cell. 2011;23(12):4446–61.

[60] Becherer KA, Rieder SE, Emr SD, Jones EW. Novel syntaxin homologue, Pep12p, required for the sorting of lumenal hydrolases to the lysosome-like vacuole in yeast. Molecular biology of the cell. 1996;7(4):579–94.

[61] Saita S, Shirane M, Natume T, Iemura SI, Nakayama KI. Promotion of neurite extension by protrudin requires its interaction with vesicle-associated membrane protein-associated protein. The Journal of biological chemistry. 2009;284(20):13766–77.

[62] Charest A, Lane K, McMahon K, Housman DE. Association of a novel PDZ domain-containing peripheral Golgi protein with the Q-SNARE (Q-soluble N-ethylmaleimide-sensitive fusion protein (NSF) attachment protein receptor) protein syntaxin 6. The Journal of biological chemistry. 2001;276(31):29456–65.

[63] Kato N, Bai H. Expression, localization and interaction of SNARE proteins in Arabidopsis are selectively altered by the dark. Plant signaling & behavior. 2010;5(11):1470–2.

[64] Zolov SN, Lupashin VV. Cog3p depletion blocks vesicle-mediated Golgi retrograde trafficking in HeLa cells. The Journal of cell biology. 2005;168(5):747–59.

[65] Dietmeier K, Honlinger A, Bomer U, Dekker PJT, Eckerskorn C, Lottspeich F, et al. Tom5 functionally links mitochondrial preprotein receptors to the general import pore. 1997;388(6638):195–200.

[66] Suzuki M, Jeong SY, Karbowski M, Youle RJ, Tjandra N. The Solution Structure of Human Mitochondria Fission Protein Fis1 Reveals a Novel TPR-like Helix Bundle. Journal of Molecular Biology. 2003;334(3):445–458.

[67] Parsons HT, Christiansen K, Knierim B, Carroll A, Ito J, Batth TS, et al. Isolation and proteomic characterization of the Arabidopsis Golgi defines functional and novel components involved in plant cell wall biosynthesis. Plant physiology. 2012;159(1):12–26.

[68] Mitoma Jy, Ito A. Mitochondrial Targeting Located at Its Carboxy Signal Terminus of Rat Liver Monoamine Oxidase B Is Targeting Signal of Monoamine Oxidase B. 1992;24:20–24.

[69] Oufattole M, Park JH, Poxleitner M, Jiang L, Rogers JC. Selective membrane protein internalization accompanies movement from the endoplasmic reticulum to the protein storage vacuole pathway in Arabidopsis. The Plant cell. 2005;17(11):3066–80.

[70] Mozdy AD, McCaffery JM, Shaw JM. Dnm1p Gtpase-Mediated Mitochondrial Fission Is a Multi-Step Process Requiring the Novel Integral Membrane Component Fis1p. The Journal of Cell Biology. 2000;151(2):367–380.

[71] Johnston AJ, Hoogenraad J, Dougan DA, Truscott KN, Yano M, Mori M, et al. Insertion and assembly of human tom7 into the preprotein translocase complex of the outer mitochondrial membrane. The Journal of biological chemistry. 2002;277(44):42197–204.

[72] Klodmann J, Sunderhaus S, Nimtz M, Jänsch L, Braun HP. Internal architecture of mitochondrial complex I from Arabidopsis thaliana. The Plant cell. 2010;22(3):797–810.

[73] Fransson S, Ruusala A, Aspenström P. The atypical Rho GTPases Miro-1 and Miro-2 have essential roles in mitochondrial trafficking. Biochemical and biophysical research communications. 2006;344(2):500–10.

[74] Latijnhouwers M, Hawes C, Carvalho C, Oparka K, Gillingham AK, Boevink P. An Arabidopsis GRIP domain protein locates to the trans-Golgi and binds the small GTPase ARL1. The Plant journal : for cell and molecular biology. 2005;44(3):459–70.

[75] Busquets A, Keim V, Closa M, del Arco A, Boronat A, Arró M, et al. Arabidopsis thaliana contains a single gene encoding squalene synthase. Plant molecular biology. 2008;67(1-2):25–36.

[76] Abeliovich H. Tlg2p, a Yeast Syntaxin Homolog That Resides on the Golgi and Endocytic Structures. Journal of Biological Chemistry. 1998;273(19):11719–11727.

[77] Carette JE, Verver J, Martens J, van Kampen T, Wellink J, van Kammen A. Characterization of plant proteins that interact with cowpea mosaic virus '60K' protein in the yeast two-hybrid system. J Gen Virol. 2002;83(4):885–893.

[78] Niihama M, Uemura T, Saito C, Nakano A, Sato MH, Tasaka M, et al. Conversion of functional specificity in Qb-SNARE VTI1 homologues of Arabidopsis. Current biology : CB. 2005;15(6):555–60.

[79] Elmore JM, Liu J, Smith B, Phinney B, Coaker G. Quantitative proteomics reveals dynamic changes in the plasma membrane during Arabidopsis immune signaling. Molecular & cellular proteomics : MCP. 2012;11(4):M111.014555.

[80] Lewis MJ, Pelham HRB. A New Yeast Endosomal SNARE Related to Mammalian Syntaxin 8. Traffic. 2002;3(12):922–929.

[81] Lapierre La, Tuma PL, Navarre J, Goldenring JR, Anderson JM. VAP-33 localizes to both an intracellular vesicle population and with occludin at the tight junction. Journal of cell science. 1999;112 (Pt 2:3723–32.

[82] Zhang ZJ, Peck SC. Simplified enrichment of plasma membrane proteins for proteomic analyses in Arabidopsis thaliana. Proteomics. 2011;11(9):1780–8.

[83] Görlich D, Rapoport Ta. Protein translocation into proteoliposomes reconstituted from purified components of the endoplasmic reticulum membrane. Cell. 1993;75(4):615–30.

[84] Chilcote TJ. Cellubrevin and synaptobrevins: similar subcellular localization and biochemical properties in PC12 cells. The Journal of Cell Biology. 1995;129(1):219–231.

[85] Kriechbaumer V, Shaw R, Mukherjee J, Bowsher CG, Harrison AM, Abell BM. Subcellular distribution of tail-anchored proteins in Arabidopsis. Traffic (Copenhagen, Denmark). 2009;10(12):1753–1764.

[86] Lee GJ, Kim H, Kang H, Jang M, Lee DW, Lee S, et al. EpsinR2 interacts with clathrin, adaptor protein-3, AtVTI12, and phosphatidylinositol-3-phosphate. Implications for EpsinR2 function in protein trafficking in plant cells. Plant physiology. 2007;143(4):1561–75.

[87] Bar-Peled M. Characterization of AtSEC12 and AtSAR1. Proteins likely involved in endoplasmic reticulum and Golgi transport. PLANT PHYSIOLOGY. 1997;114(1):315–324.

[88] Suzuki M, Youle RJ, Tjandra N. Structure of Bax. Cell. 2000;103(4):645–654.

[89] Lauber MH, Waizenegger I, Steinmann T, Schwarz H, Mayer U, Hwang I, et al. The Arabidopsis KNOLLE protein is a cytokinesis-specific syntaxin. The Journal of cell biology. 1997;139(6):1485–93.

[90] Nievergall E, Janes PW, Stegmayer C, Vail ME, Haj FG, Teng SW, et al. PTP1B regulates Eph receptor function and trafficking. The Journal of cell biology. 2010;191(6):1189–203.

[91] Rancour DM, Dickey CE, Park S, Bednarek SY. Characterization of AtCDC48. Evidence for multiple membrane fusion mechanisms at the plane of cell division in plants. Plant physiology. 2002;130(3):1241–53.

[92] Geisler M, Girin M, Brandt S, Vincenzetti V, Plaza S, Paris N, et al. Arabidopsis immunophilin-like TWD1 functionally interacts with vacuolar ABC transporters. Molecular biology of the cell. 2004;15(7):3393–405.

[93] Vallen EA. Separate domains of KAR1 mediate distinct functions in mitosis and nuclear fusion. The Journal of Cell Biology. 1992;117(6):1277–1287.

[94] Vallen EA, Scherson TY, Roberts T, van Zee K, Rose MD. Asymmetric mitotic segregation of the yeast spindle pole body. Cell. 1992;69(3):505–515.

[95] Holthuis JC, Nichols BJ, Dhruvakumar S, Pelham HR. Two syntaxin homologues in the TGN/endosomal system of yeast. The EMBO journal. 1998;17(1):113–26.

[96] Nagano M, Ihara-Ohori Y, Imai H, Inada N, Fujimoto M, Tsutsumi N, et al. Functional association of cell death suppressor, Arabidopsis Bax inhibitor-1, with fatty acid 2-hydroxylation through cytochrome b. The Plant journal : for cell and molecular biology. 2009;58(1):122–34.

[97] Kagiwada S, Hosaka K, Murata M, Nikawa J, Takatsuki A. The Saccharomyces cerevisiae SCS2 gene product, a homolog of a synaptobrevin-associated protein, is an integral membrane protein of the endoplasmic reticulum and is required for inositol metabolism. Journal of bacteriology. 1998;180(7):1700–8.

[98] Wigle JT. Molecular Cloning, Expression, and Chromosomal Assignment of Sarcolemmal-associated Proteins. A FAMILY OF ACIDIC AMPHIPATHIC alpha -HELICAL PROTEINS ASSOCIATED WITH THE MEMBRANE. Journal of Biological Chemistry. 1997;272(51):32384–32394.

[99] Cutler SR, Somerville CR. Imaging plant cell death: GFP-Nit1 aggregation marks an early step of wound and herbicide induced cell death. BMC plant biology. 2005;5:4.

[100] Chatre L, Brandizzi F, Hocquellet A, Hawes C, Moreau P. Sec22 and Memb11 are v-SNAREs of the anterograde endoplasmic reticulum-Golgi pathway in tobacco leaf epidermal cells. Plant physiology. 2005;139(3):1244–54.

[101] Saravanan RS, Slabaugh E, Singh VR, Lapidus LJ, Haas T, Brandizzi F. The targeting of the oxysterol-binding protein ORP3a to the endoplasmic reticulum relies on the plant VAP33 homolog PVA12. The Plant journal : for cell and molecular biology. 2009;58(5):817–830.

[102] Shen S, Tobery CE, Rose MD. Prm3p is a pheromone-induced peripheral nuclear envelope protein required for yeast nuclear fusion. Molecular biology of the cell. 2009;20(9):2438–50.

[103] Bennett MK, Garcia-Arrarás J, Elferink LA, Peterson K, Fleming AM, Hazuka CD, et al. The syntaxin family of vesicular transport receptors. Cell. 1993;74(5):863–873.

[104] Hwang HW, Lee JR, Chou KY, Suen CS, Hwang MJ, Chen C, et al. Oligomerization is crucial for the stability and function of heme oxygenase-1 in the endoplasmic reticulum. The Journal of biological chemistry. 2009;284(34):22672–9.

[105] Mendonsa R, Engebrecht J. Phosphatidylinositol-4,5-bisphosphate and phospholipase D-generated phosphatidic acid specify SNARE-mediated vesicle fusion for prospore membrane formation. Eukaryotic cell. 2009;8(8):1094–105.

[106] Kim DH, Eu YJ, Yoo CM, Kim YW, Pih KT, Jin JB, et al. Trafficking of phosphatidylinositol 3-phosphate from the trans-Golgi network to the lumen of the central vacuole in plant cells. The Plant cell. 2001;13(2):287–301.

[107] James DI, Parone PA, Mattenberger Y, Martinou JC. hFis1, a novel component of the mammalian mitochondrial fission machinery. The Journal of biological chemistry. 2003;278(38):36373–9.

[108] Ferro M, Salvi D, Brugière S, Miras S, Kowalski S, Louwagie M, et al. Proteomics of the chloroplast envelope membranes from Arabidopsis thaliana. Molecular & cellular proteomics : MCP. 2003;2(5):325–45.

[109] Zhang H, Kim JK, Edwards CA, Xu Z, Taichman R, Wang CY. Clusterin inhibits apoptosis by interacting with activated Bax. Nature cell biology. 2005;7(9):909–15.

[110] Kelson TL, Secor McVoy JR, Rizzo WB. Human liver fatty aldehyde dehydrogenase: microsomal localization, purification, and biochemical characterization. Biochimica et Biophysica Acta (BBA) - General Subjects. 1997;1335(1-2):99–110.

[111] Wong SH, Zhang T, Xu Y, Subramaniam VN, Griffiths G, Hong W. Endobrevin, a Novel Synaptobrevin/VAMP-Like Protein Preferentially Associated with the Early Endosome. Mol Biol Cell. 1998;9(6):1549–1563.

[112] Tang BL. Syntaxin 12, a Member of the Syntaxin Family Localized to the Endosome. Journal of Biological Chemistry. 1998;273(12):6944–6950.

[113] Katayama K, Sakurai I, Wada H. Identification of an Arabidopsis thaliana gene for cardiolipin synthase located in mitochondria. FEBS letters. 2004;577(1-2):193–8.

[114] Ishikawa F, Suga S, Uemura T, Sato MH, Maeshima M. Novel type aquaporin SIPs are mainly localized to the ER membrane and show cell-specific expression in Arabidopsis thaliana. FEBS letters. 2005;579(25):5814–20.

[115] Ito J, Batth TS, Petzold CJ, Redding-Johanson AM, Mukhopadhyay A, Verboom R, et al. Analysis of the Arabidopsis cytosolic proteome highlights subcellular partitioning of central plant metabolism. Journal of proteome research. 2011;10(4):1571–82.

[116] Loewen CJR, Roy A, Levine TP. A conserved ER targeting motif in three families of lipid binding proteins and in Opi1p binds VAP. The EMBO journal. 2003;22(9):2025–35.

[117] Advani RJ. Seven Novel Mammalian SNARE Proteins Localize to Distinct Membrane Compartments. Journal of Biological Chemistry. 1998;273(17):10317–10324.

[118] Banfield DK, Lewis MJ, Pelham HR. A SNARE-like protein required for traffic through the Golgi complex. Nature. 1995;375(6534):806–9.

[119] Mitra SK, Gantt JA, Ruby JF, Clouse SD, Goshe MB. Membrane proteomic analysis of Arabidopsis thaliana using alternative solubilization techniques. Journal of proteome research. 2007;6(5):1933–50.

[120] Steegmaier M. Three Novel Proteins of the Syntaxin/SNAP-25 Family. Journal of Biological Chemistry. 1998;273(51):34171–34179.

[121] Heath VL, Shaw SL, Roy S, Cyert MS. Hph1p and Hph2p, novel components of calcineurin-mediated stress responses in Saccharomyces cerevisiae. Eukaryotic cell. 2004;3(3):695–704.

[122] Nomura H, Komori T, Kobori M, Nakahira Y, Shiina T. Evidence for chloroplast control of external Ca2+-induced cytosolic Ca2+ transients and stomatal closure. The Plant journal : for cell and molecular biology. 2008;53(6):988–98.

[123] Dixit E, Boulant S, Zhang Y, Lee ASY, Odendall C, Shum B, et al. Peroxisomes are signaling platforms for antiviral innate immunity. Cell. 2010;141(4):668–81.

[124] Beilharz T, Egan B, Silver PA, Hofmann K, Lithgow T. Bipartite signals mediate subcellular targeting of tail-anchored membrane proteins in Saccharomyces cerevisiae. The Journal of biological chemistry. 2003;278(10):8219–23.

[125] Hwang YT, Pelitire SM, Henderson MPA, Andrews DW, Dyer JM, Mullen RT. Novel targeting signals mediate the sorting of different isoforms of the tail-anchored membrane protein cytochrome b5 to either endoplasmic reticulum or mitochondria. The Plant cell. 2004;16(11):3002–19.

[126] Dunkley TPJ, Hester S, Shadforth IP, Runions J, Weimar T, Hanton SL, et al. Mapping the Arabidopsis organelle proteome. Proceedings of the National Academy of Sciences of the United States of America. 2006;103(17):6518–23.

[127] Morandini P, Valera M, Albumi C, Bonza MC, Giacometti S, Ravera G, et al. A novel interaction partner for the C-terminus of Arabidopsis thaliana plasma membrane H + -ATPase (AHA1 isoform): site and mechanism of action on H + -ATPase activity differ from those of 14-3-3 proteins #. The Plant Journal. 2002;31(4):487–497.

[128] Ballensiefen W, Ossipov D, Schmitt HD. Recycling of the yeast v-SNARE Sec22p involves COPI-proteins and the ER transmembrane proteins Ufe1p and Sec20p. Journal of cell science. 1998;111 (Pt 1:1507–20.

[129] De Silvestris M, D'Arrigo A, Borgese N. The targeting information of the mitochondrial outer membrane isoform of cytochrome b5 is contained within the carboxyl-terminal region. FEBS letters. 1995;370(1-2):69–74.

[130] Bae MS, Cho EJ, Choi EY, Park OK. Analysis of the Arabidopsis nuclear proteome and its response to cold stress. The Plant journal : for cell and molecular biology. 2003;36(5):652–63.

[131] Burri L, Varlamov O, Doege CA, Hofmann K, Beilharz T, Rothman JE, et al. A SNARE required for retrograde transport to the endoplasmic reticulum. Proceedings of the National Academy of Sciences of the United States of America. 2003;100(17):9873–7.

[132] Hirling H. Mammalian Vesicle Trafficking Proteins of the Endoplasmic Reticulum and Golgi Apparatus. Journal of Biological Chemistry. 1996;271(10):5671–5679.

[133] Zhong W, Zhou Y, Li S, Zhou T, Ma H, Wei K, et al. OSBP-related protein 7 interacts with GATE-16 and negatively regulates GS28 protein stability. Experimental cell research. 2011;317(16):2353–63.

[134] Barbe L, Lundberg E, Oksvold P, Stenius A, Lewin E, Björling E, et al. Toward a confocal subcellular atlas of the human proteome. Molecular & cellular proteomics : MCP. 2008;7(3):499–508.

[135] Li X, Low SH, Miura M, Weimbs T. SNARE expression and localization in renal epithelial cells suggest mechanism for variability of trafficking phenotypes. American journal of physiology Renal physiology. 2002;283(5):F1111–22.

[136] Benschop JJ, Mohammed S, O'Flaherty M, Heck AJR, Slijper M, Menke FLH. Quantitative phosphoproteomics of early elicitor signaling in Arabidopsis. Molecular & cellular proteomics : MCP. 2007;6(7):1198–214.

[137] Dilcher M, Veith B, Chidambaram S, Hartmann E, Schmitt HD, Fischer von Mollard G. Use1p is a yeast SNARE protein required for retrograde traffic to the ER. The EMBO journal. 2003;22(14):3664–74.

[138] Silva PA, Ul-Rehman R, Rato C, Di Sansebastiano GP, Malhó R. Asymmetric localization of Arabidopsis SYP124 syntaxin at the pollen tube apical and sub-apical zones is involved in tip growth. BMC plant biology. 2010;10(1):179.

[139] Fransson A, Ruusala A, Aspenström P. Atypical Rho GTPases have roles in mitochondrial homeostasis and apoptosis. The Journal of biological chemistry. 2003;278(8):6495–502.

[140] Coe JG, Lim AC, Xu J, Hong W. A role for Tlg1p in the transport of proteins within the Golgi apparatus of Saccharomyces cerevisiae. Molecular biology of the cell. 1999;10(7):2407–23.

[141] von Loeffelholz O, Kriechbaumer V, Ewan RA, Jonczyk R, Lehmann S, Young JC, et al. OEP61 is a chaperone receptor at the plastid outer envelope. The Biochemical journal. 2011;438(1):143–53.

[142] Reinders J, Zahedi RP, Pfanner N, Meisinger C, Sickmann A. Toward the complete yeast mitochondrial proteome: multidimensional separation techniques for mitochondrial proteomics. Journal of proteome research. 2006;5(7):1543–54.

[143] Froehlich JE, Wilkerson CG, Ray WK, McAndrew RS, Osteryoung KW, Gage DA, et al. Proteomic study of the Arabidopsis thaliana chloroplastic envelope membrane utilizing alternatives to traditional two-dimensional electrophoresis. Journal of proteome research;2(4):413–25.

[144] Sehgal PB, Mukhopadhyay S, Xu F, Patel K, Shah M. Dysfunction of Golgi tethers, SNAREs, and SNAPs in monocrotaline-induced pulmonary hypertension. American journal of physiology Lung cellular and molecular physiology. 2007;292(6):L1526–42.

[145] Smirnova T, Stinnakre J, Mallet J. Characterization of a presynaptic glutamate receptor. Science. 1993;262(5132):430–433.

[146] Li Y, Lim S, Hoffman D, Aspenstrom P, Federoff HJ, Rempe DA. HUMMR, a hypoxia- and HIF-1alpha-inducible protein, alters mitochondrial distribution and transport. The Journal of cell biology. 2009;185(6):1065–81.

[147] Tang BL, Low DY, Lee SS, Tan AE, Hong W. Molecular cloning and localization of human syntaxin 16, a member of the syntaxin family of SNARE proteins. Biochemical and biophysical research communications. 1998;242(3):673–9.

[148] Oikawa K, Kimura S, Aoki N, Atsuta Y, Takiyama Y, Nagato T, et al. Neuronal calcium sensor protein visinin-like protein-3 interacts with microsomal cytochrome b5 in a Ca2+-dependent manner. The Journal of biological chemistry. 2004;279(15):15142–52.

[149] Stojanovski D, Koutsopoulos OS, Okamoto K, Ryan MT. Levels of human Fis1 at the mitochondrial outer membrane regulate mitochondrial morphology. Journal of cell science. 2004;117(Pt 7):1201–10.

[150] Zeng Q, Subramaniam VN, Wong SH, Tang BL, Parton RG, Rea S, et al. A novel synaptobrevin/VAMP homologous protein (VAMP5) is increased during in vitro myogenesis and present in the plasma membrane. Molecular biology of the cell. 1998;9(9):2423–37.

[151] Engel ML, Holmes-Davis R, McCormick S. Green sperm. Identification of male gamete promoters in Arabidopsis. Plant physiology. 2005;138(4):2124–33.

[152] Lewis MJ, Nichols BJ, Prescianotto-Baschong C, Riezman H, Pelham HR. Specific retrieval of the exocytic SNARE Snc1p from early yeast endosomes. Molecular biology of the cell. 2000;11(1):23–38.

[153] Hardwick KG, Pelham HR. SED5 encodes a 39-kD integral membrane protein required for vesicular transport between the ER and the Golgi complex. The Journal of cell biology. 1992;119(3):513–21.

[154] Inadome H, Noda Y, Adachi H, Yoda K. Immunoisolaton of the yeast Golgi subcompartments and characterization of a novel membrane protein, Svp26, discovered in the Sed5-containing compartments. Molecular and cellular biology. 2005;25(17):7696–710.

[155] Wagner KM, Rüegg M, Niemann A, Suter U. Targeting and function of the mitochondrial fission factor GDAP1 are dependent on its tail-anchor. PloS one. 2009;4(4):e5160.

[156] Millar AH, Eubel H, Jänsch L, Kruft V, Heazlewood JL, Braun HP. Mitochondrial cytochrome c oxidase and succinate dehydrogenase complexes contain plant specific subunits. Plant molecular biology. 2004;56(1):77–90.

[157] Burri L, Vascotto K, Gentle IE, Chan NC, Beilharz T, Stapleton DI, et al. Integral membrane proteins in the mitochondrial outer membrane of Saccharomyces cerevisiae. The FEBS journal. 2006;273(7):1507–15.

[158] Siddiqi SA, Mahan J, Siddiqi S, Gorelick FS, Mansbach CM. Vesicle-associated membrane protein 7 is expressed in intestinal ER. Journal of cell science. 2006;119(Pt 5):943–50.

[159] Latijnhouwers M, Gillespie T, Boevink P, Kriechbaumer V, Hawes C, Carvalho CM. Localization and domain characterization of Arabidopsis golgin candidates. Journal of experimental botany. 2007;58(15-16):4373–86.

[160] Shcheprova Z, Baldi S, Frei SB, Gonnet G, Barral Y. A mechanism for asymmetric segregation of age during yeast budding. Nature. 2008;454(7205):728–34.

[161] Nikolovski N, Rubtsov D, Segura MP, Miles GP, Stevens TJ, Dunkley TPJ, et al. Putative Glycosyltransferases and Other Plant Golgi Apparatus Proteins Are Revealed by LOPIT Proteomics. Plant physiology. 2012;160(2):1037–51.

[162] Mizutani M, Watanabe S, Nakagawa T, Maeshima M. Aquaporin NIP2;1 is mainly localized to the ER membrane and shows root-specific accumulation in Arabidopsis thaliana. Plant & cell physiology. 2006;47(10):1420–6.

[163] Suwastika IN, Uemura T, Shiina T, H Sato M, Takeyasu K. SYP71, a plant-specific Qc-SNARE protein, reveals dual localization to the plasma membrane and the endoplasmic reticulum in Arabidopsis. Cell structure and function. 2008;33(2):185–92.

[164] Tang BL, Low DY, Tan AE, Hong W. Syntaxin 10: a member of the syntaxin family localized to the trans-Golgi network. Biochemical and biophysical research communications. 1998;242(2):345–50.

[165] Tsuruta F, Sunayama J, Mori Y, Hattori S, Shimizu S, Tsujimoto Y, et al. JNK promotes Bax translocation to mitochondria through phosphorylation of 14-3-3 proteins. The EMBO journal. 2004;23(8):1889–99.

[166] Robinson M, Poon PP, Schindler C, Murray LE, Kama R, Gabriely G, et al. The Gcs1 Arf-GAP mediates Snc1,2 v-SNARE retrieval to the Golgi in yeast. Molecular biology of the cell. 2006;17(4):1845–58.

[167] Bernard A, Domergue F, Pascal S, Jetter R, Renne C, Faure JD, et al. Reconstitution of plant alkane biosynthesis in yeast demonstrates that Arabidopsis ECERIFERUM1 and ECERIFERUM3 are core components of a very-long-chain alkane synthesis complex. The Plant cell. 2012;24(7):3106–18.

[168] Isenmann S, Khew-Goodall Y, Gamble J, Vadas M, Wattenberg BW. A splice-isoform of vesicle-associated membrane protein-1 (VAMP-1) contains a mitochondrial targeting signal. Molecular biology of the cell. 1998;9(7):1649–60.

[169] Ferro M, Brugière S, Salvi D, Seigneurin-Berny D, Court M, Moyet L, et al. AT_CHLORO, a comprehensive chloroplast proteome database with subplastidial localization and curated information on envelope proteins. Molecular & cellular proteomics : MCP. 2010;9(6):1063–84.

[170] Low SH, Vasanji A, Nanduri J, He M, Sharma N, Koo M, et al. Syntaxins 3 and 4 are concentrated in separate clusters on the plasma membrane before the establishment of cell polarity. Molecular biology of the cell. 2006;17(2):977–89.

[171] Nechushtan a, Smith CL, Hsu YT, Youle RJ. Conformation of the Bax C-terminus regulates subcellular location and cell death. The EMBO journal. 1999;18(9):2330–41.

[172] Peltier JB, Ytterberg AJ, Sun Q, van Wijk KJ. New functions of the thylakoid membrane proteome of Arabidopsis thaliana revealed by a simple, fast, and versatile fractionation strategy. The Journal of biological chemistry. 2004;279(47):49367–83.

[173] Hatsuzawa K. Syntaxin 18, a SNAP Receptor That Functions in the Endoplasmic Reticulum, Intermediate Compartment, and cis-Golgi Vesicle Trafficking. Journal of Biological Chemistry. 2000;275(18):13713–13720.

[174] Oltvai ZN, Milliman CL, Korsmeyer SJ. Bcl-2 heterodimerizes in vivo with a conserved homolog, Bax, that accelerates programmed cell death. Cell. 1993;74(4):609–19.

[175] Seth RB, Sun L, Ea CK, Chen ZJ. Identification and characterization of MAVS, a mitochondrial antiviral signaling protein that activates NF-kappaB and IRF 3. Cell. 2005;122(5):669–82.

[176] Werhahn W. Purification and Characterization of the Preprotein Translocase of the Outer Mitochondrial Membrane from Arabidopsis. Identification of Multiple Forms of TOM20. PLANT PHYSIOLOGY. 2001;125(2):943–954.

[177] Wada Y, Nakamura N, Ohsumi Y, Hirata a. Vam3p, a new member of syntaxin related protein, is required for vacuolar assembly in the yeast Saccharomyces cerevisiae. Journal of cell science. 1997;110 (Pt 1:1299–306.

[178] Carol RJ, Breiman A, Erel N, Vittorioso P, Bellini C. PASTICCINO1 (AtFKBP70) is a nuclear-localised immunophilin required during Arabidopsis thaliana embryogenesis. Plant Science. 2001;161(3):527–535.

[179] Koch A, Yoon Y, Bonekamp NA, McNiven MA, Schrader M. A role for Fis1 in both mitochondrial and peroxisomal fission in mammalian cells. Molecular biology of the cell. 2005;16(11):5077–86.

[180] Finke K, Plath K, Panzner S, Prehn S, Rapoport TA, Hartmann E, et al. A second trimeric complex containing homologs of the Sec61p complex functions in protein transport across the ER membrane of S. cerevisiae. The EMBO journal. 1996;15(7):1482–94.

[181] Friso G, Giacomelli L, Ytterberg AJ, Peltier JB, Rudella A, Sun Q, et al. In-depth analysis of the thylakoid membrane proteome of Arabidopsis thaliana chloroplasts: new proteins, new functions, and a plastid proteome database. The Plant cell. 2004;16(2):478–99.

[182] Lister R, Chew O, Lee MN, Heazlewood JL, Clifton R, Parker KL, et al. A transcriptomic and proteomic characterization of the Arabidopsis mitochondrial protein import apparatus and its response to mitochondrial dysfunction. Plant physiology. 2004;134(2):777–89.

[183] Duncan O, Taylor NL, Carrie C, Eubel H, Kubiszewski-Jakubiak S, Zhang B, et al. Multiple lines of evidence localize signaling, morphology, and lipid biosynthesis machinery to the mitochondrial outer membrane of Arabidopsis. Plant physiology. 2011;157(3):1093–113.
