# Supplementary material for: Development of a prediction system for tail-anchored proteins
Source: BMC Bioinformatics. 2016 Sep 15;17:378. doi: 10.1186/s12859-016-1202-7 (PMC5025589; doi:10.1186/s12859-016-1202-7)
Supplement: Additional file 5 — Table S2. Initial conditional probability in the TA model. (PDF 35 kb) [file 12859_2016_1202_MOESM5_ESM.pdf]

Table S5. Unsuccessfully predicted sequences.

SP	MP	NO	total	decoding	all
AOFB_HUMAN	CSM4_YEAST	CSM4_YEAST	CSM4_YEAST	GEX2_ARATH	SEC20_YEAST
GEX2_ARATH	GEX2_ARATH	GEX2_ARATH	CYB5_HUMAN	MAVS_HUMAN	GEX2_ARATH
MAVS_HUMAN	MAVS_HUMAN	MAVS_HUMAN	GEX2_ARATH	MTX1_HUMAN	TOM7_HUMAN
MIRO1_HUMAN	MTX1_HUMAN	MTX1_HUMAN	MAVS_HUMAN	O22825_ARATH	YD012_YEAST
MIRO2_HUMAN	O22825_ARATH	O22825_ARATH	MIRO2_HUMAN	PEX15_YEAST	PGC1_YEAST
MTX1_HUMAN	O80952_ARATH	O80952_ARATH	MTX1_HUMAN	PGC1_YEAST	MAVS_HUMAN
NDB3B_ARATH	PGC1_YEAST	PGC1_YEAST	NDB3B_ARATH	Q9FNB2_ARATH	MTX1_HUMAN
O22825_ARATH	Q93YW7_ARATH	Q93YW7_ARATH	O22825_ARATH	Q9M1X3_ARATH	TOM7_YEAST
PGC1_YEAST	Q9FNB2_ARATH	Q9FNB2_ARATH	O80952_ARATH	SEC20_YEAST	O22825_ARATH
Q93YW7_ARATH	Q9SKK2_ARATH	Q9SKK2_ARATH	PGC1_YEAST	TLG2_YEAST	Q9FNB2_ARATH
Q9C7H2_ARATH	SEC20_YEAST	SEC20_YEAST	Q93YW7_ARATH	TOM22_YEAST	
Q9FNB2_ARATH	TO221_ARATH	TO221_ARATH	Q9FNB2_ARATH	TOM5_ARATH	
Q9SKK2_ARATH	TOM6_YEAST	TOM6_YEAST	Q9SKK2_ARATH	TOM6_YEAST	
SEC20_YEAST	TOM7_HUMAN	TOM7_HUMAN	Q9SLI4_ARATH	TOM7_HUMAN	
TLG2_YEAST	TOM7_YEAST	TOM7_YEAST	SEC20_YEAST	TOM7_YEAST	
TO221_ARATH	YD012_YEAST	YBM6_YEAST	TLG2_YEAST	YD012_YEAST	
TOM7_HUMAN		YD012_YEAST	TO221_ARATH		
TOM7_YEAST			TOM6_YEAST		
YBM6_YEAST			TOM7_HUMAN		
YD012_YEAST			TOM7_YEAST		
			YBM6_YEAST		
			YD012_YEAST		

Each column corresponds to an evaluation method. The "SP","MP", "NO", and "total" columns list sequences with failed likelihood score-based predictions in the SP, MP, NO, and entire negative sets, respectively. Sequences that did not generate proper topology are indicated in the "decoding" column. Sequences that failed in all methods are indicated in the all column.
