# Supplementary material for: Development of a prediction system for tail-anchored proteins
Source: BMC Bioinformatics. 2016 Sep 15;17:378. doi: 10.1186/s12859-016-1202-7 (PMC5025589; doi:10.1186/s12859-016-1202-7)
Supplement: Additional file 6 — Table S3. Initial conditions for the SP model. (PDF 35 kb) [file 12859_2016_1202_MOESM6_ESM.pdf]

Table S3. Initial conditions for the SP model.

Amino acid	Output probability					
	Signal peptide			Cap region	Globular region	TMD
	1-6	7-15	16-40			
A	0.02941	0.06667	0.06122	0.02941	0.05	0.08753
C	0.02941	0.02222	0.02041	0.02941	0.05	0.04376
D	0.02941	0.02222	0.08163	0.02941	0.05	0.00656
E	0.02941	0.02222	0.14286	0.02941	0.05	0.00656
F	0.02941	0.02222	0.08163	0.02941	0.05	0.13129
G	0.02941	0.11111	0.02041	0.02941	0.05	0.08753
H	0.02941	0.02222	0.02041	0.02941	0.05	0.02188
I	0.02941	0.11111	0.02041	0.02941	0.05	0.13129
K	0.02941	0.02222	0.08163	0.23529	0.05	0.00656
L	0.08824	0.17778	0.02041	0.02941	0.05	0.13129
M	0.14706	0.02222	0.02041	0.02941	0.05	0.06565
N	0.02941	0.02222	0.08163	0.02941	0.05	0.01094
P	0.02941	0.02222	0.02041	0.02941	0.05	0.02188
Q	0.02941	0.02222	0.08163	0.02941	0.05	0.01094
R	0.02941	0.02222	0.02041	0.23529	0.05	0.00656
S	0.14706	0.02222	0.02041	0.02941	0.05	0.01094
T	0.02941	0.02222	0.08163	0.02941	0.05	0.01094
V	0.14706	0.15556	0.08163	0.02941	0.05	0.13129
W	0.02941	0.06667	0.02041	0.02941	0.05	0.06565
Y	0.02941	0.02222	0.02041	0.02941	0.05	0.01094

The SP region is further divided into 3 sections, corresponding to areas from the starting point to the 6th residue, from the 7th to 15th residue, and from the 16th to 40th residue.
