# Supplementary material for: Development of a prediction system for tail-anchored proteins
Source: BMC Bioinformatics. 2016 Sep 15;17:378. doi: 10.1186/s12859-016-1202-7 (PMC5025589; doi:10.1186/s12859-016-1202-7)
Supplement: Additional file 7 — Table S4. Initial conditions for the MP model. (PDF 37 kb) [file 12859_2016_1202_MOESM7_ESM.pdf]

Table S4. Initial conditions for the MP model.

Amino acid	Output probability						
	Signal peptide			Cap region	Globular region	TMD	Loop
	1-6	7-15	16-40				
A	0.02941	0.06667	0.06122	0.02941	0.05405	0.08753	0.02381
C	0.02941	0.02222	0.02041	0.02941	0.05405	0.04376	0.02381
D	0.02941	0.02222	0.08163	0.02941	0.05405	0.00656	0.11905
E	0.02941	0.02222	0.14286	0.02941	0.05405	0.00656	0.11905
F	0.02941	0.02222	0.08163	0.02941	0.05405	0.13129	0.02381
G	0.02941	0.11111	0.02041	0.02941	0.05405	0.08753	0.02381
H	0.02941	0.02222	0.02041	0.02941	0.05405	0.02188	0.02381
I	0.02941	0.11111	0.02041	0.02941	0.02703	0.13129	0.02381
K	0.02941	0.02222	0.08163	0.23529	0.05405	0.00656	0.02381
L	0.08824	0.17778	0.02041	0.02941	0.02703	0.13129	0.02381
M	0.14706	0.02222	0.02041	0.02941	0.05405	0.06565	0.02381
N	0.02941	0.02222	0.08163	0.02941	0.05405	0.01094	0.11905
P	0.02941	0.02222	0.02041	0.02941	0.05405	0.02188	0.02381
Q	0.02941	0.02222	0.08163	0.02941	0.05405	0.01094	0.11905
R	0.02941	0.02222	0.02041	0.23529	0.05405	0.00656	0.02381
S	0.14706	0.02222	0.02041	0.02941	0.05405	0.01094	0.07143
T	0.02941	0.02222	0.08163	0.02941	0.05405	0.01094	0.07143
V	0.14706	0.15556	0.08163	0.02941	0.02703	0.13129	0.02381
W	0.02941	0.06667	0.02041	0.02941	0.05405	0.06565	0.02381
Y	0.02941	0.02222	0.02041	0.02941	0.05405	0.01094	0.07143

The initial output probability conditions of the SP region, cap region, and TMD region were identical to those in the SP model. In the globular region, the initial output probabilities for hydrophobic residues frequently observed in TMDs (I, L, and V) were set low. In the loop region, the initial conditions were set such that some hydrophobic residues (I, V, and L) were less likely to occur, whereas some hydrophilic and acidic residues (D, E, N, and Q) were more likely to occur.
